# Exercise, physical activity, and self-determination theory: A systematic review

**DOI:** 10.1186/1479-5868-9-78

**Published:** 2012-06-22

**Authors:** Pedro J Teixeira, Eliana V Carraça, David Markland, Marlene N Silva, Richard M Ryan

**Affiliations:** 1 Faculty of Human Kinetics, Technical University of Lisbon, Estrada da Costa, 1495-688, Cruz Quebrada, Portugal; 2 School of Sport, Health and Exercise Sciences, Bangor University, Bangor, UK; 3 Department of Clinical and Social Sciences in Psychology, University of Rochester, Rochester, NY, USA

## Abstract

**Background:**

Motivation is a critical factor in supporting sustained exercise, which in turn is associated with important health outcomes. Accordingly, research on exercise motivation from the perspective of self-determination theory (SDT) has grown considerably in recent years. Previous reviews have been mostly narrative and theoretical. Aiming at a more comprehensive review of empirical data, this article examines the empirical literature on the relations between key SDT-based constructs and exercise and physical activity behavioral outcomes.

**Methods:**

This systematic review includes 66 empirical studies published up to June 2011, including experimental, cross-sectional, and prospective studies that have measured exercise causality orientations, autonomy/need support and need satisfaction, exercise motives (or goal contents), and exercise self-regulations and motivation. We also studied SDT-based interventions aimed at increasing exercise behavior. In all studies, actual or self-reported exercise/physical activity, including attendance, was analyzed as the dependent variable. Findings are summarized based on quantitative analysis of the evidence.

**Results:**

The results show consistent support for a positive relation between more autonomous forms of motivation and exercise, with a trend towards identified regulation predicting initial/short-term adoption more strongly than intrinsic motivation, and intrinsic motivation being more predictive of long-term exercise adherence. The literature is also consistent in that competence satisfaction and more intrinsic motives positively predict exercise participation across a range of samples and settings. Mixed evidence was found concerning the role of other types of motives (e.g., health/fitness and body-related), and also the specific nature and consequences of introjected regulation. The majority of studies have employed descriptive (i.e., non-experimental) designs but similar results are found across cross-sectional, prospective, and experimental designs.

**Conclusion:**

Overall, the literature provides good evidence for the value of SDT in understanding exercise behavior, demonstrating the importance of autonomous (identified and intrinsic) regulations in fostering physical activity. Nevertheless, there remain some inconsistencies and mixed evidence with regard to the relations between specific SDT constructs and exercise. Particular limitations concerning the different associations explored in the literature are discussed in the context of refining the application of SDT to exercise and physical activity promotion, and integrating these with avenues for future research.

## Introduction

Physical activity and exercise, when undertaken regularly, are highly beneficial for health, and for physical and psychological well-being [e.g., [1]. Yet, only a minority of adults in modern societies reports engaging in physical exercise at a level compatible with most public health guidelines [2]. For instance, 2009 data indicate that, on a typical week, 60% of adults in Europe engaged in *no* physical exercise or sports [3]. In the US, less than 50% of adults are considered regularly physically active [4] while in Canada new accelerometer data shows that only 15% of adults meet national physical activity recommendations [5]. Such findings suggest that many people lack sufficient motivation to participate in the 150 minutes of moderately intense exercise or physical activity^a^ per week recommended [6]. Indeed, approximately 40% of Europeans agree with the statement: “Being physically active does not really interest me – I would rather do other things with my spare time” [3].

Lack of motivation can broadly be explained by two orders of factors. First, as highlighted in the previous statistic, people may not be sufficiently interested in exercise, or value its outcomes enough to make it a priority in their lives [7]. Many individuals experience competing demands on their time from educational, career, and family obligations, possibly at the expense of time and resources that could be invested in exercising regularly. Second, some people may not feel sufficiently competent at physical activities, feeling either not physically fit enough or skilled enough to exercise, or they may have health limitations that present a barrier to activity [8]. Whether it be low interest or low perceived competence, the physical activity participation data indicate that many people are either unmotivated (or *amotivated*), having no intention to be more physically active, or are insufficiently motivated in the face of other interests or demands on their time.

In addition to those who are unmotivated, another source of short-lived persistence in exercise behaviors comes from people who do express personal motivation to exercise regularly, yet initiate exercise behaviors with little follow through. Specifically, a significant percentage of people may exercise because of *controlled* motivations, where participation in activities like going to the gym or running regularly is based on a feeling of “having to” rather than truly “wanting to” participate [7]. Controlled forms of motivation, which by definition are not *autonomous* (i.e., they lack volition), are predominant when the activity is perceived primarily as a means to an end and are typically associated with motives or goals such as improving appearance or receiving a tangible reward [9]. One hypothesis then is that the stability of one’s motivation is at least partially dependent on some of its qualitative features, particularly the degree of perceived autonomy or of an *internal perceived locus of causality*[10]. That is, the level of reflective self-endorsement and willingness associated with a behavior or class of behaviors should be associated with greater persistence. An utilitarian approach to exercise (and to exercise motivation), such as might be prevalent in fitness clubs or other settings where exercise is externally prescribed, could thus be partially responsible for the high dropout rate observed in exercise studies [e.g., [11]. In fact, the pervasiveness of social and medical pressures toward weight loss, combined with externally prescriptive methods may be ill-suited to promote sustained increases in population physical activity levels.

In sum, large numbers of individuals are either unmotivated or not sufficiently motivated to be physically active, or are motivated by types of externally-driven motivation that may not lead to sustained activity. This highlights the need to look more closely at goals and self-regulatory features associated with regular participation in exercise and physical activity. Self-determination theory (SDT) is uniquely placed among theories of human motivation to examine the differential effects of qualitatively different types of motivation that can underlie behavior [12]. Originating from a humanistic perspective, hence fundamentally centered on the fulfillment of needs, self-actualization, and the realization of human potential, SDT is a comprehensive and evolving macro-theory of human personality and motivated behavior [12]. In what follows we will briefly describe key concepts formulated within SDT (and tested in SDT empirical studies) that are more relevant to physical activity and exercise, all of which will be implicated in our empirical review.

First, SDT distinguishes between intrinsic and extrinsic types of motivation regulating one’s behavior. *Intrinsic motivation* is defined as doing an activity because of its inherent satisfactions. When intrinsically motivated the person experiences feelings of enjoyment, the exercise of their skills, personal accomplishment, and excitement [13]. To different degrees, recreational sport and exercise can certainly be performed for the associated enjoyment or for the challenge of participating in an activity. In contrast to intrinsic motivation, *extrinsic motivation* refers to doing an activity for instrumental reasons, or to obtain some outcome separable from the activity *per se*. For example, when a person engages in an activity to gain a tangible or social reward or to avoid disapproval, they are extrinsically motivated. SDT, however, conceptualizes qualitatively different types of extrinsic motivation, that themselves differ in terms of their relative autonomy. Some extrinsic motives are relatively heteronomous, representing what in SDT are described as *controlled* forms of motivation. For example, externally regulated behaviors are those performed to comply with externally administered reward and punishment contingencies. Also controlled are extrinsic motivations based on introjected regulation, where behavior is driven by self-approval. Controlled forms of extrinsic motivation are expected within SDT to sometimes regulate (or motivate) short-term behavior, but not to sustain maintenance over time [14]. Yet not all extrinsic motives are controlled. When a person does an activity not because it is inherently fun or satisfying (intrinsic motivation), but rather because it is of personal value and utility, it can represent a more autonomous form of behavioral regulation. Specifically in SDT*,* identified and integrated forms of behavioral regulation are defined as those in which one’s actions are self-endorsed because they are personally valued. Examples include exercising because one values its outcomes and desires to maintain good health [7]. Thus, in SDT, these different forms of motivation are conceptualized as lying along a continuum from non-autonomous to completely autonomous forms of behavioral regulation.

Third, SDT introduces the concept of *basic psychological needs* as central to understanding both the satisfactions and supports necessary for high quality, autonomous forms of motivation*.* Specifically SDT argues that there are basic psychological needs for autonomy, competence, and relatedness, all of which are conceived as essential and universal nutriments to psychological health and the development of internal motivation. Satisfaction of these basic needs results in increased feelings of vitality and well-being [15]. Like any other activity, engaging in sports and exercise can be more or less conducive to having one’s psychological needs realized [16]. For example, experiences of competence vary upon success or failure at challenging physical tasks or as a function of feedback from, for example, a fitness professional. Perceptions of personal connection (relatedness) with others (e.g., fellow members of a fitness class or weight loss program) can vary greatly as a function of the interpersonal environment. Feelings of autonomy (versus feeling controlled) differ as a function of communication styles in exercise settings. According to SDT, in fact, need fulfillment in any context is closely associated with the characteristics of that social milieu, that is, whether important others support the needs for autonomy (e.g., take the perspective of the client/patient, support their choices, minimize pressure), relatedness (e.g., create an empathetic and positive environment, show unconditional regard), and competence (e.g., limit negative feedback, provide optimally challenging tasks). The concept of *need support* is thus thought to largely explain individual differences in the development and enactment of motivation across the lifespan [12]. Consequently, the design of health behavior change interventions that enhance satisfaction of participants’ basic needs is a matter of much interest in SDT studies, including in the area of exercise and physical activity [17,18].

More recently, *goal contents* have also been explored from an SDT perspective in relation to a range of behaviors, including exercise [e.g., [19,20]. It should be noted that most authors have referred to goal contents in exercise contexts as *motives*, or more specifically *participation motives* [e.g., [64,79]. Operationally both terms are identical and we will use them interchangeably herein. Whereas intrinsic motivation and the various forms of extrinsic motivation represent the regulatory processes underlying a behavior, motives or goal contents are the outcomes that individuals are pursuing by engaging in the behavior [12]. Goal contents are differentiated according to the extent to which their pursuit is likely to satisfy basic psychological needs. Specifically, SDT distinguishes *intrinsic goals* (e.g., seeking affiliation, personal growth, or health) as those thought to be more closely related to the fulfillment of basic psychological needs, from *extrinsic goals* (e.g., seeking power and influence, wealth, or social recognition) that are thought to be associated with “substitute needs” which are neither universal nor truly essential to well-being and personal development. Factor analytic studies have borne out this theoretical distinction, and a number of studies have shown the predicted differential consequences of intrinsic versus extrinsic goal importance [21,22]. Within the domain of exercise and physical activity, extrinsic goals (e.g., when exercise is performed primarily to improve appearance) or intrinsic goals (e.g., to challenge oneself or to improve/preserve health and well-being) can clearly be distinguished. It should be noted that different goals or motives towards a given activity often naturally co-exist in the same person, some being more intrinsic, some less. Similar to what occurs with motivational regulations (which can have more or less autonomous elements, see more below), it is the relative preponderance of certain types of motives versus others which is thought to determine more or less desirable outcomes [e.g., [19,20].

Finally, SDT also proposes that people have dispositional tendencies, named *causality orientations*[14] which describe the way they preferentially orient towards their environments, resulting in characteristic motivational and behavioral patterns. Although some people may be more inclined to seek out and follow their internal indicators of preference in choosing their course of action, others may more naturally tend to align with external directives and norms, while still others may reveal to be generally amotivated, more passive, and unresponsive to either internal or external events that could energize their actions [12]. Although this topic has not been explored at length in previous research, these orientations can manifest themselves (and be measured) in exercise and physical activity contexts and the *Exercise Causality Orientation Scale* has been developed to measure individual differences in orientations around exercise [9].

**Figure 1:**
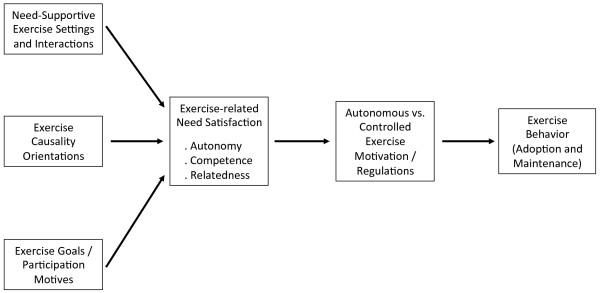
**General SDT process model for exercise behavior.** Adapted from the general health process model (Ref Ryan et al., Europ Health Psych, 2009), this graph includes the 5 groups of variables analyzed in this review as exercise predictors and their expected relationships (in a simplified version). Although this review only covers direct relationships between each class of variables (e.g., need satisfaction in exercise) and exercise behaviors, since few articles have simultaneously tested various steps of this model, the SDT model for exercise assumes that a sizable share of variance of exercise associated with SDT variables may be explained via indirect or mediating mechanisms, as depicted. See Discussion for more details.

Previous review papers of the topic of SDT and physical activity have primarily focused on describing the rationale for the application of this particular theoretical framework to physical activity behaviors, reviewing illustrative studies [7,23,24]. Meanwhile, the SDT-related exercise empirical research base has grown considerably in recent years, warranting a more comprehensive and systematic review of empirical data. Systematic reviews and meta-analyses of empirical studies provide the highest level of evidence for the appraisal and synthesis of findings from scientific studies. Accordingly, the present review includes 66 empirical studies published up to June 2011 that assessed relations between SDT-based constructs or interventions and exercise outcomes. We included experimental and cross-sectional studies that have measured exercise causality orientations, autonomy/need support and need satisfaction, exercise motives or goals, and exercise self-regulations and motivation. We also studied SDT-based interventions as predictors of exercise behavioral outcomes. Figure 1 depicts a general model of SDT and exercise, where its major constructs and theoretical links are highlighted.

## Methods

### Data sources and procedure

This review is limited to articles written in English and published in peer-reviewed journals covering adult samples. Research on autonomy and exercise in adolescents and children (typically based in school and physical education) was excluded, as well as studies with competitive athletic samples. Both are specific settings and were considered distinct from leisure-time or health-related exercise participation in adults, the focus of this review. The review includes both cross-sectional and longitudinal studies, investigating clinical and/or general population samples, and using diverse quantitative methodological approaches. A systematic literature search of studies published between 1960 and June 2011 was undertaken on the computerized psychological and sport databases PsycINFO and SportDiscus. The following strategy was used: TX (autonomous motivation OR autonomous regulation OR intrinsic motivation OR controlled regulation OR autonomy OR self-determination OR treatment regulations OR goals OR motives OR basic needs OR autonomy-supportive climate) AND TX (physical activity OR exercise OR exercise behavior OR leisure-time physical activity) Limiters were: Scholarly (peer-reviewed) journals; English Language; Adulthood (> 18 yr); Specific subjects: exercise OR motivation OR self-determination. This search yielded 660 articles. Abstracts were read and, of those, all potentially relevant full manuscripts were retrieved (n = 73). At this stage, studies were excluded which did not include either SDT variables or physical activity variables (accounting for most of the excluded studies), that used non-adult samples, and that reported achievement/performance outcomes related to PE classes. Next, reference lists of retrieved articles, previous review articles on the topic, and books were also reviewed, and manual searches were conducted in the databases and journals for authors who regularly publish in this area. This search yielded 11 additional manuscripts, totaling 84 potentially relevant manuscripts. Next, manuscripts were read and the following inclusion criteria used to select the final set of manuscripts: inclusion of non-athletic samples; outcomes included exercise/physical activity behaviors; reported direct associations between self-determination variables and physical activity outcomes. A total of 66 studies fulfilled all inclusion criteria and thus were included in this review. Of these, ten were experimental, eleven prospective, forty-two cross-sectional, and three used mixed designs.

Studies were initially coded with a bibliography number, but independent samples (*K*) were considered as the unit of analysis in the current review since a few studies used the same sample while other studies reported analyses on multiple samples. Data tables (Table 1) were constructed and encompassed sample characteristics of study populations, motivational predictors of exercise behavior, instruments of assessment, exercise-related outcomes, research designs, and statistical methods used to test the associations.

**Table 1:** Description of reviewed studies

**Reference**	**Design**	**Sample**			**Measures**	**Significant Predictors**		**Outcomes**	**Analysis/Observations**
		**Size (%F)**	**Features**	**Location**					
***I. Exercise self-regulations and related measures***									
ThØgersen-Ntoumani & Ntoumanis, 2006 [52]	Cross-sectional	375 (51)	Exercisers (Mean 38.7 yr)	UK	Exercise self-regulations (BREQ) + amotivation (AMS)	*MV*: IM (+) ^a^, ID (+) ^a,b^, INTR (+) ^a^; EXT (−) ^a,b^, AMOT (−) ^a^		Exercise stages of change ^a^; Exercise relapses (fewer) ^b^	Multivariate logistic regressions, adjusting for sex and age; Manovas
Rose et al., 2005 [56]	Cross-sectional	184 (55)	Healthy adults (17–60 yr)	UK	Exercise self-regulations (BREQ)	*MV*: IM (+) ^a^, ID (+) , INTR (+) EXT (−)		Exercise stages of change	Discriminant function analysis (IM was redundant); Manovas ^a^
Ingledew et al., 2009 [79]	Cross-sectional	251 (52)	University Students (Mean 19.5 yr)	UK	Exercise self-regulations (BREQ-2)	*MV*: IM (+),ID (+),INTR (n.s) EXT (n.s)		Self-reported exercise (measure analogous to LTEQ)	Partial Least Squares Analysis (PLS); Mediation analysis
Edmunds et al., 2006 [44]	Cross-sectional	369 (52)	Healthy individuals (Mean 31.9 yr)	UK	Exercise self-regulations (BREQ)	*MV*: IM (n.s.), ID (+), INTR (+), EXT (−)		Self-reported exercise (total and strenuous PA; LTEQ)	Multiple regressions; Mediation analysis. No associations with mild/moderately intense PA.
						*BIV*: IM (+), ID (+), INTR (+), EXT (n.s.)			
Wilson et al., 2006 [85]	Cross-sectional	139 (64)	Undergraduate students (Mean 19.5 yr)	Canada	Exercise extrinsic self-regulations (BREQ) and Integrated Regulation scale (INTEG)	*MV*: INTEG (+), ID (+), INTR (+), EXT (n.s.)		Self-reported exercise (LTEQ)	Bivariate correlations; Multiple regression analysis
						*BIV*: INTEG (+), ID (n.s.), INTR (n.s.), EXT (n.s.)			
McDonough et al., 2007 [50]	Cross-sectional	558 (72)			Exercise self-regulations (BREQ)	*MV*: RAI (+)		Self-reported exercise (LTEQ)	Bivariate correlations; SEM; Mediation analysis. Only RAI was tested in multivariate analysis.
						*BIV*: RAI (+), IM (n.s.), ID (+), INTR (n.s.), EXT (n.s.)			
Daley & Duda, 2006 [55]	Cross-sectional	409 (61)	Undergraduate students (19.9 yr)	UK	Exercise self-regulations (BREQ-2)	*MV*: IM (+), ID (++), INTR (+); EXT (− M); AMOT (− F)		Exercise stages of change; Physical activity status (from inactive to active)	Discriminant function analysis
Wilson et al., 2004 [45]	Cross-sectional	276 (64)	Undergraduate students (20.5 yr)	Canada	Exercise self-regulations (BREQ-2)	*MV*: IM (n.s.); ID (+), INTR (+ F; - M), EXT (n.s.), AMOT (n.s.)		Self-reported exercise (LTEQ)	Bivariate correlations; Multiple regressions analysis
						*BIV*: IM (+); ID (+), INTR (+ F), EXT (n.s.), AMOT (n.s.)			
Markland, 2009 [9]	Cross-sectional	102 F	Healthy individuals (Mean 29.2 yr)	UK	Exercise self-regulations (BREQ-2)	*MV*: IM (+), ID (+), AMOT (n.s.)		Self-reported exercise (LTEQ)	Bivariate correlations; Multiple regression/mediation (Preacher & Hayes): INTR and EXT not analyzed.
						*BIV*: IM (+), ID (+), INTR (+), EXT (n.s.), AMOT (−)			
Ingledew & Markland, 2008 [46]	Cross-sectional	252 (48)	Office workers (Mean 40 yr)	UK	Exercise self-regulations (BREQ-2)	*MV*: IM (n.s.), ID (+), INTR (n.s.), EXT (−)		Self-reported exercise (measure analogous to LTEQ)	Bivariate correlations; SEM
						*BIV*: IM (+), ID (+), INTR (n.s.), EXT (−)			
Peddle et al., 2008 [43]	Cross-sectional	413 (46)	Colorectal cancer survivors (Mean 60 yr)	Canada	Exercise self-regulations (BREQ-2)	*MV*: IM (n.s.), ID (+), INTR (+), EXT (n.s.), AMOT (n.s.)		Self-reported exercise (LTEQ)	Bivariate correlations; Path analysis
						*BIV*: IM (+), ID (+), INTR (+), EXT (n.s.), AMOT (−)			
Landry & Solmon, 2004 [86]	Cross-sectional	105 F	African-American (Mean 56 yr)	USA	Exercise self-regulations (BREQ)	*MV*: IM (+), ID (+), INTR (−), EXT (n.s.)		Exercise stages of change; exercise categories	Anovas; Discriminant function analysis
						*BIV*: RAI (+); IM (+), ID (+), INTR (n.s.), EXT (n.s.)			
Milne et al., 2008 [87]	Cross-sectional	558 F	Breast cancer survivors (Mean 59 yr)	Australia	Exercise self-regulations (BREQ-2)	*MV*: IM (+), ID (+), INTR (n.s.), EXT (n.s.), AMOT (n.s.)		Self-reported exercise (LTEQ); exercise categories (meeting vs. not meeting guidelines)	Anovas; Hierarchical regression analysis
						*BIV*: IM (+), ID (+), INTR (n.s.), EXT (−), AMOT (−)			
Mullan & Markland, 1997 [57]	Cross-sectional	314 (49.7)	Healthy individuals (Mean 35–40 yr)	UK	Exercise self-regulations (BREQ)	*MV*: IM (+), ID (+), INTR (n.s.), EXT (n.s.)		Exercise stages of change	Anova (RAI was analyzed); Discriminant function analysis;
						*BIV*: RAI (+)			
Lewis & Sutton, 2011 [48]	Cross-sectional	100 (50)	95% undergraduates, members of a university gym; age not specified	UK	Exercise self-regulations (BREQ-2)	*MV*: IM (+); ID (n.s.), INTR (n.s.), EXT (−), AMOT (n.s.)		Exercise frequency	Bivariate correlations; Multiple regression analysis
						*BIV*: IM (+); ID (+), INTR (+), EXT (−), AMOT (−)			
Markland & Tobin, 2010 [88]	Cross-sectional	133 F	Exercise referral scheme clients (Mean 54.5 yr)	UK	Exercise self-regulations (BREQ-2)	*BIV*: IM (+), ID (+), INTR (n.s.), EXT (n.s.), AMOT (n.s.)		Self-reported exercise (LTEQ)	Bivariate correlations
Wilson et al., 2002 [49]	Cross-sectional	500 (81)	Aerobic exercisers (Mean 34 yr)	Canada	Exercise self-regulations (BREQ)	*BIV*: IM (+), ID (+), INTR (+), EXT (−)		Self-reported exercise (LTEQ)	Bivariate correlations. Differences between PA intensities.
Sebire et al., 2009 [19]	Cross-sectional	410 (71)	Exercisers (Mean 41.4 yr)	UK	Exercise self-regulations (BREQ)	*MV*: RAI (+)		Self-reported exercise (LTEQ)	Bivariate correlations; Hierarchical regression analysis
						*BIV*: RAI (+)			
Brickell & Chatzisarantis, 2007 [42]	Cross-sectional	252 (61)	College students (Mean 23.2 yr)	Canada	Exercise self-regulations (BREQ)	*MV*: IM (n.s.), ID (+), INTR (n.s.), EXT (n.s)		Self-reported exercise (LTEQ)	Multiple regression analysis
						*BIV*: IM (+), ID (+), INTR (+), EXT (n.s)			
Edmunds et al., 2006 [51]	Cross-sectional	339 (53)	Symptomatic vs asymptomatic for exercise dependence (Mean 32.1 yr)	UK	Exercise self-regulations (BREQ) and Integrated Regulation scale (INTEG)	*MV*: Symptomatic: INTR (+ tendency); Asymptomatic: ID (+). Remaining variables not significant.		Self-reported exercise (total and strenuous PA; LTEQ)	Multiple regressions. No associations with moderately intense PA.
Moreno et al., 2007 [89]	Cross-sectional	561 (53)	Healthy adults (Mean 31.8 yr)	Spain	Exercise self-regulations (BREQ-2)	*MV*: IM (n.s.), ID (−), INTR (n.s.), EXT (−), AMOT (−)		Exercise duration (0-45 min vs. 45-60 min vs. > 60 min)	Manovas
Hall et al., 2010 [90]	Cross-sectional	470 (54)	Adults (Mean 44.9 yr)	Canada	Exercise self-regulations (BREQ-2); Self-reported exercise (LTEQ)	*BIV*: IM (+), ID (+), INTR (+), EXT (n.s.), AMOT (−)		Exercise status (active vs. inactive)	Anovas
Standage et al., 2008 [91]	Cross-sectional	52 (50)	University students (Mean 22 yr)	UK	Exercise self-regulations; Autonomous and controlled motivations (BREQ)	*MV*: AutMot (+), CtMot (n.s.) BIV: IM (+), ID (+), INTR (n.s.), EXT (n.s), AutMot (+), CtMot (n.s.)		Accelerometry	Bivariate correlations; Sequential regression analysis
Duncan et al., 2010 [41]	Cross-sectional	1079 (57)	Regular exercisers (Mean 24.2 yr)	Canada	Exercise self-regulations (BREQ-2) + Integrated reg. scale	*MV*: IM (n.s.), INTEG (+), ID (+)*, INTR (n.s.), EXT (n.s), AMOT (n.s)		* PA frequency; PA intensity; PA duration (LTEQ)	Bivariate correlations; Multiple regression analysis
						*BIV*: IM (+), INTEG (+), ID (+), INTR (+), EXT (− F)*, AMOT (−)			
Sorensen et al. 2006 [54]	cross-sectional	109 (59)	Psychiatric patients (Mean age group 31–49 yr)	Norway	Exercise regulations (based on BREQ)	*MV*: IM (+), ID (n.s.), INTR (n.s.), EXT (n.s.)		Self-reported exercise level	Bivariate correlations; Logistic regressions
						*BIV*: IM (+), ID (n.s.), INTR (n.s.), EXT (−)			
Puente & Anshel, 2010 [77]	Cross-sectional	238 (57)	College students (Mean 20.4 yr)	USA	Exercise self-regulations (SRQ-E)	*MV*: RAI (+)		Exercise frequency	Bivariate correlations; SEM
						*BIV*: RAI (+)			
Halvary et al., 2009 [76]	Cross-sectional	190 (44)	Healthy adults (Mean 21.8 yr)	Norway	Autonomous motivation (SRQ)	*MV*: AutMot (+)		Exercise frequency and duration	Bivariate correlations; SEM; Mediation analysis
						*BIV*: AutMot (+)			
Wilson et al., 2006 [29]	Cross-sectional	220; 220 (56)	Cancer survivors (Mean 60–64 yr) vs non-cancer (Mean 50 yr)	Canada	Autonomous and controlled motivation (TSRQ-PA)	*MV*: AutMot (+), CtMot (−) in both samples		Self-reported exercise (min/wk of MVPA)	Bivariate correlations; Multiple regression analysis
						*BIV*: AutMot (+), CtMot (n.s.) in both samples			
Hurkmans et al., 2010 [92]	Cross-sectional	271 (66)	Patients with Rheumatoid Arthritis (Mean 62 yr)	Netherlands	Exercise self-regulations (TSRQ-PA). Adated RAI.	*MV*: RAI (+)		Self-reported exercise (SQUASH)	Bivariate correlations; Multiple regression analysis
						*BIV*: RAI (+)			
Lutz et al., 2008 [93]	Cross-sectional	535 (60)	University students (Mean 20 yr)	USA	Exercise self-regulations (EMS). Adapted RAI.	*MV*: RAI (+)		Self-reported exercise (LTEQ)	Bivariate correlation; Preacher & Hayes mediation analysis
						*BIV*: RAI (+)			
Wininger, 2007 [28]	Cross-sectional	143; 58 (76)	Undergraduates (Mean 21–22 yr)	USA	Exercise self-regulations (EMS)	*MV* *: IM (+), INTEG (+), ID (+), INTR (+), EXT (n.s.), AMOT (−)		* Exercise stages of change; ** Distance walked on treadmill	Bivariate correlations; Manovas
						*BIV* **: IM experience sensations (+), INTEG (n.s.), ID (n.s.), INTR (n.s.), EXT (n.s.), AMOT (−)			
Craike, M., 2008 [47]	Cross-sectional	248 (53)	Healthy adults (Mean 48 yr)	Australia	Exercise self-regulations (based on BREQ and EMS)	*MV*: IM (+), ID (n.s.), INTR (n.s.), EXT (−)		Self-reported LTPA	SEM
Tsorbatzoudis et al., 2006 [94]	Cross-sectional	257 (55)	Healthy adults (Mean 31 yr)	Greece	Exercise self-regulations (SMS)	*MV*: IM (+), ID (+), INTR (+), EXT (−), AMOT (−)		Exercise frequency (from the least to the most frequent)	Multivariate analysis of variance; multiple regressions
Chatzisarantis & Biddle, 1998 [95]	Cross-sectional	102 (50)	University employees (Mean 40 yr)	UK	Behavioral regulations for PA (SMS adaptation)	*MV*: Autonomous group (vs controlled) based on RAI scores (+)		Self-reported exercise (LTEQ)	SEM
Matsumoto & Takenaka, 2004 [96]	Cross-sectional	486 (53)	Healthy individuals (Mean 45 yr)	Japan	Exercise self-regulations (SDMS); profiles of self-determination	*BIV*: IM (+), ID (+), INTR (+), EXT (n.s.) AMOT (−); Self-determined profile (+)		Exercise stages of change	Bivariate correlations and cluster analysis
McNeill et al., 2006 [97]	Cross-sectional	910 (80)	Healthy individuals (Mean 33 yr)	USA	Intrinsic and extrinsic motivations (MPA)	*MV*: Intrinsic motivation (+); Extrinsic motivation for social pressure		Self-reported exercise (minutes of walking, and MVPA)	SEM. Indirectly through self-efficacy.
Russell & Bray, 2009 [98]	Cross-sectional and prospective (6 + 6wk)	68 (13)	Cardiac rehabilitation outpatients (Mean 64.9 yr)	Canada	Exercise self-regulations (BREQ-2)	*MV*: RAI (+)		Self-reported exercise (7Day-PAR)	Bivariate correlations; Multiple regression analysis
						*BIV*: RAI (+)			
Russell & Bray, 2010 [99]	Cross-sectional and Observational (14wk)	53 M	Exercise cardiac rehabilitation patients (Mean 62.8 yr)	Canada	Exercise self-regulations (SRQ-E)	*MV*: AutMot (+)		Exercise frequency; duration (+); volume (+) – 7Day-PAR	Bivariate correlations; Hierarchical regression analysis
						*BIV*: AutMot (+), CtMot (n.s.)			
Fortier et al., 2009 [100]	Prospective (6mo)	149 F	Healthy adults (Mean 51.8 yr)	Canada	Exercise self-regulations (TSRQ-adapted)	*MV*: AutMot (n.s.)		Duration, Frequency, and Energy Expenditure (CHAMPS)	Bivariate correlations; Mediation/regression analysis
						*BIV*: AutMot (n.s.), CtMot (n.s.)			
Rodgers et al., 2010 [31]	Prospective	1572 (60)	Initiate vs. long-term exercisers (Mean 22–51 yr)	Canada	Exercise self-regulations (BREQ)	*MV*: IM (+), ID (+), INTR (n.s.), EXT (−) overtime for initiates, but < to regular exercisers		Self-reported exercise (LTEQ); Initiate vs. long-term exercisers	Manovas. Total N from 6 samples: initiates (60, 134, 38, 84), regular exercisers (202, 1054)
Barbeau et al., 2009 [101]	Prospective (1mo)	118 (65)	Healthy adults (Mean 19 yr)	Canada	Exercise self-regulations (BREQ-2)	*MV*: AutMot (+), CtMot (n.s.)		Self-reported exercise (LTEQ)	Bivariate correlations; Path analysis
						*BIV*: AutMot (+), CtMot (n.s.)			
Hagger et al., 2006 [35]	Prospective (4wk)	261 (64)	University students (Mean 24.9 yr)	UK	Relative autonomy index (based on PLOC scale)	*BIV*: RAI (+)		Self-reported exercise (frequency)	Bivariate correlations; SEM
Hagger et al., 2006 [34]	Prospective (4 wk)	261 (64)	Exercise sample of university students (Mean 24.9 yr)	UK	Relative autonomy index (based on PLOC Scale)	*BIV*: RAI (+)		Self-reported exercise (frequency)	Bivariate correlations
Kwan et al., 2011[53]	Prospective (4 wk)	104 (58)	Undergraduate students; active (Mean 18.2 yr)	USA	Exercise self-regulations (BREQ-2)	*BIV*: IM (+), ID (n.s.), INTR (n.s.), EXT (n.s.), AMOT (n.s.), RAI (n.s)		Self-reported exercise (online diary)	Bivariate correlations
Edmunds et al., 2007 [38]	Prospective (uncontrolled intervention) (3mo)	49 (84)	Overweight/Obese patients (Mean BMI: 38.8; Mean 45 yr) on an exercise scheme	UK	Exercise self-regulations (BREQ-2); Integrated regulation subscale (EMS)	*MV*: IM (n.s.), INTEG (+), ID (−)*, INTR (+)*, EXT (n.s.)		Self-reported exercise (LTEQ);	Bivariate correlations; Multilevel regression analysis.* ID and INTR multivariate outcomes resulted from net suppression; thus, not considered by the authors.
						*BIV*: ID (+), INTR (−)			
Wilson et al., 2003 [58]	Experimental (12wk)	53 (83)	Adults (Mean 41.8 yr; BMI: 19.9 ± 3.0 kg/m^2^)	Canada	Exercise self-regulations (BREQ)	*MV*: IM (+), ID (+)		Self-reported exercise (LTEQ)	Bivariate correlations; Multiple regression analysis. IM and ID increased from pre- to post-exercise program
						*BIV*: IM (+), ID (+), INTR (n.s.), EXT (n.s.)			
Sweet et al., 2009 [102]	Experimental (12mo)	234 (38)	Inactive with type 2 diabetes (Mean 53 yr) on an exercise program	Canada	Exercise self-regulations (BREQ)	*MV*: AutMot (+)		Amount of PA (kcal/month)	Bivariate correlations; Regression/Mediation analysis
						*BIV*: AutMot (+)			
Fortier et al., 2011 [36]	Experimental (13wk); RCT	120 (69)	Inactive patients (Mean 47.3 yr): intensive vs. brief PA intervention	Canada	Exercise self-regulations (BREQ-2)	*BIV*: IM, ID, INTR, EXT, and RAI were not significant predictors		Self-reported exercise (LTEQ)	Bivariate correlations
Fortier et al., 2007 [17]	Experimental (13wk); RCT	120 (69)	Autonomy supportive vs brief PA counseling (Mean 47.3 yr)	Canada	Treatment self-regulations (TSRQ-PA)	*MV*: AutMot (+)		Self-reported exercise (LTEQ)	Bivariate correlations; Path/Mediation analysis
						*BIV*: AutMot (n.s.)			
Levy & Cardinal, 2004 [40]	Experimental (2mo); RCT	185 (68)	Adults (Mean 46.8 yr); SDT-based mail intervention vs. controls	USA	Exercise self-regulations (EMS)	*MV*: IM, INTEG, ID, INTR, EXT, and AMOT were not significant predictors		Self-reported exercise (LTEQ)	Manovas with repeated measures
Mildestvedt et al., 2008 [68]	Experimental (4wk); RCT	176 (22)	Cardiac rehabilitation patients (Mean 56 yr): SDT-based vs standard rehab treatment	Norway	Autonomous and controlled motivations (TSRQ)	*BIV*: AutMot (+); CtMot (n.s.)		Self-reported exercise (composite score); exercise intensity	ANOVAs with repeated measures
Silva et al., 2010 [33]	Experimental (12mo); RCT	239 F	OW/Obese women (Mean 38 yr); SDT-treatment vs controls	Portugal	Exercise self-regulations (SRQ-E)	MV: IM (+)*, ID (n.s.), INTR (n.s.), EXT (n.s.)		Self-reported exercise: MVPA * (7-day PAR); lifestyle PA index	Bivariate correlations; PLS analysis; Mediation analysis
						BIV: IM (+), ID (+), INTR (+), EXT (n.s.)			
Silva et al., 2010 [32]	Experimental (1 yr + 2y follow-up); RCT	221 F	OW/Obese women (Mean 38 yr); SDT-treatment vs controls	Portugal	Exercise self-regulations (SRQ-E) at 1 yr and 2 yr	MV: AutMot 2 yr (+), INTR 2 yr (n.s.), EXT 2 yr (n.s.)		2-yr self-reported exercise: MVPA (7-day PAR)	Bivariate correlations; PLS analysis; Mediation analysis
						BIV: AutMot 1 and 2 yr (+), INTR 2 yr (+), EXT 2 yr (n.s.)			
***II. Exercise-related psychological need satisfaction***									
Puente & Anshel, 2010 [77]	Cross-sectional	238 (57)	College students (Mean 20.4 yr)	USA	Basic Psychological Needs Scale (BPNS); Perceived Competence Scale (PCS)	*MV*: Competence (+)		Exercise frequency	Bivariate correlations; SEM; Relatedness not measured.
						*BIV*: Autonomy (n.s.), Competence (+)			
Edmunds et al., 2006 [44]	Cross-sectional	369 (52)	Healthy individuals (Mean 31.9 yr)	UK	Psychological need satisfaction (BNSWS adapted)	*MV*: Autonomy (n.s.), Competence (+), Relatedness (n.s.)		Self-reported exercise (total and strenuous PA; LTEQ)	Bivariate correlations; Regression analysis; mediation analysis
						*BIV*: Autonomy (+), Competence (+), Relatedness (+)			
Edmunds et al., 2006 [51]	Cross-sectional	339 (53)	Symptomatic vs asymptomatic for exercise dependence (Mean 32.1 yr)	UK	Psychological need satisfaction (BNSWS adapted)	*BIV*: Autonomy (n.s.), Competence (+), Relatedness (n.s.)		Self-reported exercise (total and strenuous PA; LTEQ)	Bivariate correlations. No associations with mild/moderately intense PA
Peddle et al., 2008 [43]	Cross-sectional	413 (46)	Colorectal cancer survivors (Mean 60 yr)	Canada	Psychological need satisfaction (PNSE)	*BIV*: Autonomy (+), Competence (+), Relatedness (+)		Self-reported exercise (LTEQ)	Bivariate correlations
McDonough et al., 2007 [50]	Cross-sectional	558 (72)	Recreational dragon boat paddlers (Mean 45 yr)	Canada	Exercise need satisfaction (PNSE)	*MV*: Autonomy (−), Competence (+)		Self-reported exercise (LTEQ)	Bivariate correlations; SEM
						*BIV*: Autonomy (n.s.), Competence (+), Relatedness (n.s.)			
Sebire et al., 2009 [19]	Cross-sectional	410 (71)	Exercisers (Mean 41.4 yr)	UK	Exercise need satisfaction (PNSE)	*BIV*: Exercise need satisfaction (composite score) (+)		Self-reported exercise (LTEQ)	Bivariate correlations
Milne et al., 2008 [87]	Cross-sectional	558 F	Breast cancer survivors (Mean 59 yr)	Australia	Perceived competence (PCS)	*MV*: Competence (+)		Self-reported exercise (LTEQ); Exercise categories (meeting vs. not meeting guidelines)	Anovas; Hierarchical regression analysis
						*BIV*: Competence (+)			
Halvary et al., 2009 [76]	Cross-sectional	190 (44)	Healthy adults (Mean 21.8 yr)	Norway	Perceived competence (PCS)	*MV*: Competence (n.s.)		Exercise frequency and duration	Bivariate correlations; SEM/Mediation analysis
						*BIV*: Competence (+)			
Vlachopoulos & Michailidou, 2006 [103]	Cross-sectional	508 (50)	Greek adults (Mean 30 yr)	Greece	Psychological needs satisfaction in exercise (BPNES)	*MV*: Autonomy (n.s.), Competence (+); Relatedness (n.s.)		Exercise frequency	SEM
Markland & Tobin, 2010 [88]	Cross-sectional	133 F	Exercise referral scheme clients	UK	Autonomy need (LCE); Perceived Competence (IMI); Relatedness (8-item scale)	*BIV*: Autonomy (+), Competence (+), Relatedness (n.s.)		Self-reported exercise (LTEQ)	Bivariate correlations
Russell & Bray, 2009 [98]	Cross-sectional and prospective (6 + 6wk)	68 (13)	Cardiac rehabilitation outpatients (Mean 64.9 yr)	Canada	Exercise need satisfaction (PNSE)	*BIV*: Autonomy (n.s.), Competence (+)*, Relatedness (n.s.)		Self-reported exercise (7Day-PAR) at 3wk and 6wk* follow-up	Bivariate correlations
Barbeau et al., 2009 [101]	Prospective (1mo)	118 (65)	Healthy adults (Mean 19 yr)	Canada	Exercise need satisfaction (PNSES)	*BIV*: Autonomy (+), Competence (+), Relatedness (+)		Self-reported exercise (LTEQ)	Bivariate correlations
Hagger et al., 2006 [34]	Prospective (4 wk)	261 (64)	Exercise sample of university students (Mean 24.9 yr)	UK	Psychological need satisfaction	*BIV*: Psychological need satisfaction (composite score) (+)		Self-reported exercise (frequency).	Bivariate correlations
Edmunds et al., 2007 [38]	Prospective (uncontrolled intervention) (3mo)	49 (84)	OW/Obese patients (BMI: 38.75; Mean 45 yr)	UK	Psychological need satisfaction (PNSS)	*MV*: Autonomy (n.s.), Competence (n.s.); Relatedness (n.s.)		Self-reported exercise (LTEQ); (Increase in relatedness overtime)	Multilevel regression analysis; Paired T-tests
Fortier et al., 2007 [17]	Experimental (13 wk); RCT	120 (69)	Healthy adults (Mean 47.3 yr)	Canada	Perceived Competence (PCES)	*MV*: Competence (+)		Self-reported exercise (LTEQ)	Path analysis; Mediation analysis
Levy & Cardinal, 2004 [40]	Experimental (2mo); RCT	185 (68)	Adults (Mean 46.8 yr); SDT-based mail intervention vs. controls	USA	Perceived autonomy satisfaction (LCE)	*MV*: Autonomy (+ F), Competence (n.s.), Relatedness (n.s.)		Self-reported exercise (LTEQ)	Manovas with repeated measures
Silva et al., 2010 [33]	Experimental (12mo); RCT	239 F	OW/Obese women (Mean BMI: 31.5; Mean 38 y); SDT-based weight loss treatment vs controls	Portugal	Perceived autonomy satisfaction (LCE); Competence (IMI)	*BIV*: Autonomy (+), Competence (+)		Self-reported exercise: MVPA (7-day PAR); lifestyle PA index	Bivariate correlations
***III. Exercise motives and related measures***									
Ingledew et al., 2009 [79]	Cross-sectional	251 (52)	University Students (Mean 19.5 yr)	UK	Exercise motives (EMI-2)	*MV*: Intrinsic motives: Stress management (+), Affiliation (+), Challenge (+); Extrinsic: Health/fitness (+); body-related (n.s.)		Self-reported exercise (measure analogous to LTEQ)	Partial Least Squares Analysis (PLS); Mediation analysis
Ingledew & Markland, 2008 [46]	Cross-sectional	252 (48)	Office workers (Mean 40 yr)	UK	Exercise motives (EMI-2)	*BIV*: Intrinsic motives (n.s.), Extrinsic motives: health/fitness (+) and body-related (−)		Self-reported exercise (measure analogous to LTEQ)	Bivariate correlations
Frederick & Ryan, 1993 [59]	Cross-sectional	376 (64)	Healthy individuals (Mean 39 yr)	USA	Exercise motives (MPAM)	Intrinsic motives: interest/enjoyment (+); competence (+); Extrinsic motives: body-related (+)		Self-reported exercise (levels, intensity)	Differences between PA categories; correlations and Manovas
Frederick et al., 1996 [104]	Cross-sectional	118 (68)	College students (Mean 22 yr)	USA	Exercise motives (MPAM-r)	*MV*: Extrinsic: body-related (+ M)		Self-reported exercise: frequency, volume	Bivariate correlations; Multiple regression analysis
						*BIV*: Intrinsic motives (+ F), Extrinsic: body-related (+ M)			
Buckworth et al., 2007 [30] a	Cross-sectional	184;220 (60)	University students (Mean 18–22 yr)	USA	Exercise motives (EMI and IMI; total and subscales)	Intrinsic motives (except choice) (+); Extrinsic motives (except tangible rewards) (+)		Exercise stages of change	Anovas and profile analysis
Sebire et al., 2009 [19]	Cross-sectional	400 (73)	Exercisers (Mean 41.4 yr)	UK	Exercise goal content (GCEQ)	*MV*: Intrinsic motives (+)		Self-reported exercise (LTEQ)	Bivariate correlations; Hierarchical regression analysis
						*BIV*: Intrinsic motives (+)			
Segar et al., 2006 [64]	Cross-sectional	59 F	Healthy adults (Mean 45.6 yr)	USA	Body and non-body shape motives for exercise (via inductive, qualitative methods)	*BIV*: Body motives (−); non-body shape motives (+).		Self-reported exercise (LTEQ)	Hierarchical regression analysis
Sit et al., 2008 [105]	Cross-sectional	360 F	Chinese adults (30–59 yr)	China	Exercise motives (MPAM-r)	*MV*: Intrinsic motives : competence/challenge (+), interest/enjoyment (+); Extrinsic: fitness/health (+); appearance (n.s.)		Exercise stages of change	Manovas
Davey et al., 2009 [106]	Cross-sectional	134 (66)	Employees (estimated mean age between 25–44 yr)	New Zeland	Exercise motives (based on MPAM-r and SMS)	*MV*: Intrinsic motives: enjoyment (+), competence/challenge (+); Extrinsic: appearance (−); Fitness (n.s.)		Total number of steps in 3wk	Multiple regression analysis
Segar et al., 2008 [65]	Prospective	156 F	Healthy women (Mean 49.3 yr)	USA	Extrinsic and Intrinsic goals (based on a list of goals and on cluster analysis)	*MV*: Intrinsic goals (+); Extrinsic goals: weight maintenance/toning (−); health benefits (−)		Self-reported exercise (LTEQ)	Linear mixed model
Ingledew et al., 1998 [107]	Prospective (3mo)	425 (34)	Government employees (Mean 40 yr)	UK	Exercise motives (EMI-2)	*MV*: Intrinsic motives: enjoyment (+); Extrinsic: body-related (+ action; - maintenance); health pressures (+ preparation; - action/maintenance)		Exercise stages of change	Discriminant function analysis
Ryan et al., 1997 [27] a	Prospective (10wk)	40 (80)	University students and employees (Mean 21 yr)	USA	Exercise motives (MPAM)	*MV*: Intrinsic motives: enjoyment (+), competence (+); body-related motives (n.s.)		Reduced dropout and attendance to exercise classes	Manovas and multiple regressions
Ryan et al., 1997 [27] b	Prospective (10wk)	155 (57)	New fitness center members (Mean 19.5 yr)	USA	Exercise motives (MPAM-R)	*MV*: Intrinsic motives: enjoyment (+), competence (+), social interactions (+); Extrinsic motives: fitness (n.s.), appearance (n.s.)		Attendance to and duration of exercise workout	Manovas and multiple regressions
Buckworth et al., 2007 [30] b	Experimental (10wk)	142 (66)	College Students (Mean 21.3 yr)	USA	Exercise motives (EMI and IMI);	*BIV*: Intrinsic motives: effort/competence (+) and interest/enjoyment (+); Extrinsic motives: appearance (+) *		Exercise patterns (from stable inactive to stable active); Activity vs. Lecture (no activity) Classes *	Anovas with repeated measures.
***IV. Perceived need support***									
Peddle et al., 2008 [43]	Cross-sectional	413 (46)	Colorectal cancer survivors (Mean 60 yr)	Canada	Perceived need support (PAS, based on HCCQ-short)	*BIV*: Need support (+)		Self-reported exercise (LTEQ)	Bivariate correlations
Milne et al., 2008 [87]	Cross-sectional	558 F	Breast cancer survivors (Mean 59 yr)	Australia	Perceived need support (mHCCQ)	*MV*: Need support (+) *BIV*: Need support (+)		Self-reported exercise (LTEQ); exercise categories (meeting vs. not meeting guidelines)	Anovas; Hierarchical regression analysis
Hurkmans et al., 2010 [92]	Cross-sectional	271 (66)	Patients with Rheumatoid Arthritis (Mean 62 yr)	Netherlands	Perceived need support (HCCQ-mod)	*MV*: Need support (n.s.)		Self-reported PA (SQUASH)	Bivariate correlations; Multiple regression analysis
						*BIV*: Need support (n.s.)			
Halvary et al., 2009 [76]	Cross-sectional	190 (44)	Healthy adults (Mean 21.8 yr)	Norway	Perceived need support (SCQ based on HCCQ)	*BIV*: Need support (+)		Exercise frequency and duration	Bivariate correlations
Markland & Tobin, 2010 [88]	Cross-sectional	133 F	Exercise referral scheme clients	UK	Need support (15-item scale)	*BIV*: Need support (n.s.)		Self-reported exercise (LTEQ)	Bivariate correlations
Puente & Anshel, 2010 [77]	Cross-sectional	238 (57)	College students (Mean 20.4 yr)	USA	Exercise need support (SCQ)	*BIV*: Need support (+)		Exercise frequency	Bivariate correlations
Russel & Bray, 2010 [99]	Cross-sectional and prospective (14wk)	53 M	Exercise cardiac rehabilitation patients (Mean 62.8 yr)	Canada	Perceived need support (HCCQ-short)	*MV*: Need support (n.s.)		Exercise frequency; duration (+); volume – 7Day-PAR	Bivariate correlations; Hierarchical regression analysis
						*BIV*: Need support (+)			
Levy et al., 2008 [108]	Prospective (8-10wk)	70 (37)	Injured exercisers in rehabilitation (Mean 33 yr; 69% recreational)	UK	Perceived need support (HCCQ)	*MV*: Need support (+) ^a, c^		Exercise adherence: ^a^ clinical, ^b^ home-based; ^c^ attendance	Bivariate correlations; Manovas
						*BIV*: Need support (+) ^a, c^			
Edmunds et al., 2007 [38]	Uncontrolled Prospective (3mo)	49 (84)	OW/Obese patients (BMI: 38.75; Mean 45 yr) on an exercise scheme	UK	Perceived need support (HCCQ)	*MV*: Need support (n.s.)		Self-reported exercise (LTEQ);	Multilevel regression analysis
Fortier et al., 2007 [17]	Experimental (13 wk); RCT	120 (69)	Autonomy supportive vs. brief PA counseling (Mean 47.3 yr)	Canada	Perceived need support (HCCQ)	*BIV*: Need support		Self-reported exercise (LTEQ)	Bivariate correlations
Mildestvedt et al., 2008 [68]	Experimental (4wk); RCT	176 (22)	Cardiac rehabilitation patients (Mean 56 yr): autonomy supportive vs. standard rehab	Norway	Perceived need support (mHCCQ)	*MV*: Need support (n.s.)		Self-reported exercise (composite score); exercise intensity	Manovas with repeated measures
Silva et al., 2010 [33]	Experimental (12mo); RCT	239 F	OW/Obese women (Mean BMI: 31.5; Mean 38 y): SDT-based WL treatment vs. controls	Portugal	Perceived need support (HCCQ)	*MV*: Need support (+)		Self-reported exercise: MVPA (7-day PAR); lifestyle PA index	Bivariate correlations; PLS/mediation analysis
						*BIV*: Need support (+)			
Silva et al., 2010 [32]	Experimental (1 yr + 2y follow-up); RCT	221 F	OW/Obese women (Mean BMI: 31.5; Mean 38 y): SDT-based WL treatment vs. controls	Portugal	Perceived need support (HCCQ)	*BIV*: Need support (+)		Self-reported exercise: MVPA (7-day PAR)	Bivariate correlations
***V. Exercise Causality Orientations***									
Rose et al., 2005 [56]	Cross-sectional	375 (51)	Volunteers (17–60 yr)	UK	Exercise causality orientations (ECOS)	*MV*: Autonomy O. (+), Controlling O. (− F), and Impersonal O. (−)		Exercise stages of change	Discriminant function analysis. Gender differences
Kwan et al., 2011[53]	Prospective (4 wk)	104 (58)	Undergraduate students; active (Mean 18.2 yr)	USA	Exercise causality orientations (ECOS)	*BIV*: Autonomy O. (+), Controlling O. (−), and Impersonal O. (n.s.)		Self-reported exercise (online diary)	Bivariate correlations
***VI. SDT-based Interventions and other SDT-related measures***									
Edmunds et al., 2008 [39]	Experimental (10wk)	55 F	Exercisers (Mean 21 yr)	UK	Exercise self-regulations (BREQ-2); Need support (PESS); Basic needs (PNSS); Exercise attendance	Groups: SDT-based exercise classes vs. traditional exercise classes	Higher perceived need support, autonomy and relatedness needs; Competence (+), INTRO (+) and amotivation (−) overtime for both groups	Higher exercise attendance	Multilevel regression analysis
Fortier et al., 2007 [17]	Experimental (13wk); RCT	120 (69)	Healthy adults (Mean 47.3 yr)	Canada	Exercise self-regulations (TSRQ-PA); Perceived Competence (PCES); Need Support (HCCQ); Self-reported exercise (LTEQ)	Groups: autonomy supportive vs. brief PA counseling	Higher perceived need support, autonomous motivation overtime	Higher reported exercise overtime	Ancovas
Fortier et al., 2011 [36]	Experimental (13wk); RCT	120 (69)	Inactive primary care patients (Mean 47.3 yr): intensive vs. brief PA counseling intervention	Canada	Exercise self-regulations (BREQ-2); Self-reported exercise (LTEQ)	Groups: autonomy supportive - intensive vs. brief PA counseling	Higher perceived need support, autonomous motivation overtime	Higher reported exercise overtime	Ancovas
Mildestvedt et al., 2008 [68]	Experimental (4wk); RCT	176 (22)	Cardiac rehabilitation patients (Mean 56 yr): autonomy supportive vs. standard rehab	Norway	Exercise self-regulations (TSRQ); Perceived need support (mHCCQ); Self-reported exercise	Groups: autonomy supportive vs. standard rehab	No significant differences	No significant differences	Anovas with repeated measures
Levy & Cardinal, 2004 [40]	Experimental (2mo); RCT	185 (68)	Adults (Mean 46.8 yr); SDT-based mail intervention vs. controls	USA	Exercise self-regulations (EMS); Perceptions of autonomy (LCE); Competence (PSPP); Self-reported exercise (LTEQ)	Groups: SDT-based mail vs. controls	Women only: increase in perception of autonomy	Women only: increase self-reported exercise	Anovas with repeated measures
Silva et al., 2010 [18]	Experimental (12mo); RCT	239 F	OW/Obese women (Mean BMI: 31.5; Mean 38 y); RCT	Portugal	Exercise self-regulations (SRQ-E); Need support (HCCQ); Perceived autonomy (LCE); Self-reported exercise (MVPA, lifestyle, steps)	Groups: SDT-based weight loss treatment vs. controls	Higher need supportive climate, autonomy satisfaction, IM, IDENT, INTRO	Higher reported exercise (all measures)	Effect sizes; T-tests
Silva et al., 2011 [32]	Experimental (1 yr + 2y follow-up); RCT	221 F	OW/Obese women (Mean BMI: 31.5; Mean 38 y); RCT	Portugal	Exercise self-regulations (SRQ-E) at 1 yr and 2 yr; Need support (HCCQ); Self-reported exercise (MVPA)	Groups: SDT-based weight loss treatment vs. controls	Higher 2-yr EXT, INTRO and autonomous regulations	Higher 2-yr reported exercise	Effect sizes; T-tests

Legend: F, female; M, male ; BIV, uni/bivariate associations; MV, multivariate associations; IM, intrinsic motivation; INTEG, integrated regulation; ID, identified regulation; INTR, introjected regulation; EXT, external regulation; AMOT, amotivation; RAI, relative autonomy index; AutMot, autonomous motivations; CtMot, controlled motivations; Autonomy O., autonomy orientation; Controlling O., controlling orientation; Impersonal O., impersonal orientation; (+), positive association; (-), negative association; (n.s.), not significant. Superscript letters are used to signal associations between specific predictors and outcomes (check the ‘significant predictors’ and ‘outcomes’ columns when applied). (*) is used when specific comments need to be made (check the ‘observations’ column on those cases).

### Organization of SDT predictors

Studies were generally organized based on the self-determination theory process model, depicted in Figure 1. The goal of the present manuscript was not to test this model *per se*, which would involve a considerably larger analysis. Instead, we focused exclusively on relations between each of these categories of variables and exercise outcomes (described below). Results concerning exercise self-regulations are listed first, followed by findings reporting the association between psychological needs satisfaction and exercise behavioral outcomes. Next, results concerning the measures of exercise motives/goals are reported, followed by findings regarding the association between perceived need support and exercise. Exercise causality orientation studies are listed last. In addition, we also identified interventions based on SDT and analyzed their effects on exercise outcomes.

### Exercise-related outcomes

Exercise behavior was evaluated through self-reported measures (e.g., *7-day Physical Activity Recall* (PAR) [25], *Godin Leisure-Time Exercise Questionnaire* (LTEQ) [26]) in a total of 55 independent samples (78%). Three studies (representing 4 original samples) used accelerometry or pedometry to measure physical activity (6%). Measures of stages of change for exercise participation were employed in 13 samples (18%). A few other indicators were also used in some cases (8%), namely exercise attendance, number of exercise relapses, and exercise dropout.

### Data coding and analyses

Summary tables were created based on the analysis of the available data (Tables 2 and 3). Sample characteristics (i.e., sample size, age, gender) were summarized using a tallying system and resulted in total counts (see Table 2). The percentage of independent samples presenting each characteristic from the total number of samples was also included. A summary of the evidence for each SDT-based construct was determined through a calculation of the percentage of independent samples supporting each association, based on whether the association was statistically significant or not (see Table 3). In all studies, significance level was set at 0.05. The measures of association varied across the studies’ statistical methods, as indicated in the column “observations” in Table 1, including correlation and multiple regression coefficients, *t*-test or ANOVA group differences (e.g., between active and inactive groups), discriminant function coefficients, and structural equation model path coefficients, among others. Because many studies included bivariate associations (or direct paths in structural models) and also multivariate associations (in regression or in structural models), these were analyzed separately (see Table 2). A sum code was built for each motivational construct based on the following classification system: Positive (++) for percentage *K* ≥75% and (+) for percentage *K* between 50-75% showing positive associations in both bivariate and multivariate tests; 0/+ or 0/- when the evidence was split between no association (0) and either positive or negative associations, respectively; and (?) for other results indicating inconsistent findings or indeterminate results due to a small number of studies available).

**Table 2:** Summary of samples characteristics

**Characteristics**	**Samples*K*(%)**
Sample size	
< 100	13 (18.0)
100-300	38 (52.8)
300-500	12 (16.7)
≥ 500	9 (12.5)
Gender	
Women only	11 (15.3)
Men only	1 (1.4)
Men and Women – Combined	46 (63.9)
Men and Women – Separately	14 (19.4)
Location	
Western countries	70 (97.2)
Non-western countries	2 (2.8)
Mean age, years	
≤24	21 (29.2)
25-44	28 (38.8)
45-64	22 (30.6)
≥ 65	1 (1.4)
Design	
Cross-Sectional	45 (62.5)
Longitudinal – Observational	16 (22.2)
Longitudinal – Experimental	9 (12.5)
Mixed Method	2 (2.8)
Exercise Data Collection	
Self-reported Exercise	56 (77.8)
Exercise Stages of Change	13 (18.1)
Accelerometry/pedometry	4 (5.6)
Other*	6 (8.3)
Total *K*	72

Note: *Exercise relapses, weekly attendance, exercise adherence (home; clinical), exercise dropout.

**Table 3:** Summary of associations between SDT predictors and exercise-related outcomes

			**%** ***K*Supporting associations**			
**Predictors**	**# of S*tudies***	***K***	**+**	**-**	**0**	**Sum code**
Exercise Regulations/Motivations						
Intrinsic motivation	26 (22)	37 (24)	62 (92)	0 (0)	38 (8)	**+**
Integrated regulation	6 (3)	8 (4)	62 (75)	0 (0)	38 (25)	**+**
Identified regulation	27 (24)	38 (26)	74 (85)	2 (0)	24 (15)	**+**
Introjected regulation	26 (25)	37 (27)	30 (52)	5 (4)	65 (44)	**0/+**
External regulation	26 (24)	37 (26)	0 (0)	43 (23)	57 (77)	**0/-**
Amotivation	10 (11)	14 (13)	0 (0)	36 (69)	64(31)	**0/-**
Relative autonomy (e.g., RAI)	8 (13)	8 (12)	88 (83)	0 (0)	12 (17)	**++**
Autonomous regulations	10 (10)	11 (11)	91 (82)	0 (0)	9 (18)	**++**
Controlled regulations	4 (6)	5 (7)	0 (0)	60 (0)	40 (100)	**0/-**
Need-Supportive Climate	6 (11)	6 (11)	50 (73)	0 (0)	50 (27)	**+**
Psychological Needs in Exercise						
Autonomy	4 (9)	5 (10)	20 (50)	20 (0)	60 (50)	**0/+**
Competence	8 (12)	9 (13)	56 (92)	0 (0)	44 (8)	**+**
Relatedness	4 (7)	4 (8)	0 (38)	0 (0)	100 (62)	**0**
Composite score*	0 (2)	0 (2)	0 (100)	0 (0)	0 (0)	**?**
Exercise Motives/Goals						
Intrinsic	7 (5)	8 (8)	100 (75)	0 (0)	0 (25)	**++**
Health/fitness	6 (1)	6 (1)	33 (100*)	33 (0)	33 (0)	**?**
Body-related	7 (5)	8 (8)	25 (63)	25 (12)	50 (25)	**0/+**
Exercise Causality Orientations						
Autonomy*	1 (1)	2 (1)	100 (100)	0 (0)	0 (0)	**?**
Controlling*	1 (1)	2 (1)	0 (0)	50 (100)	50 (0)	**?**
Impersonal*	1 (1)	2 (1)	0 (0)	100 (0)	0 (100)	**?**

Legend: Results derived from multivariate analyses and uni/bivariate analyses (in parenthesis) are presented. *K*, number of samples. Positive (++) was used for percentage *K* ≥75% and (+) for percentage *K* between 50-75% for both bivariate and multivariate associations; 0/+ or 0/- when the evidence was split between no association (0) and either positive or negative associations, respectively; (?) for other results indicating inconsistent findings or indeterminate results (i.e., when only a small number of studies were available, marked with *).

## Results

### Characteristics of studies and samples

The 66 located studies comprised a total of 72 independent samples. The number of samples was higher than the total number of studies because some studies analyzed data originating from more than one sample (two samples: [27], [28], [29]; three samples: [30]; six samples: [31]). On the other hand, 7 studies were published using data from three original samples ([18,33,32]; [35,34]; [17,36]). A summary of the demographic characteristics of participants and samples is reported in Table 2. Samples tended to be mixed gender and included a range of populations (e.g., healthy individuals, chronic disease patients, overweight/obese individuals, exercisers), predominantly from Western cultures (97%), and mainly aged between 25–65 years-old.

From the studies eligible for this review, 53 (*K* = 57) analyzed associations between self-regulations and exercise behavioral outcomes, 17 studies (*K* = 17) investigated the relations between basic psychological needs and exercise, 12 studies (*K* = 15) tested the associations between motives and exercise, and 13 studies (*K* = 12) included measures of perceived need support and evaluated its predictive effect on exercise-related outcomes (see Table 3). Seven intervention studies, corresponding to 6 actual interventions, were identified. It should be noted that relations reported in the intervention studies were also analyzed in the other sections (e.g., regulations, need support, etc.)

### Motivational predictors of exercise-related outcomes

*Exercise behavioral regulations.* A total of 57 samples (53 studies) analyzed associations between regulations and exercise behavior. Of these, 37 were used in cross-sectional designs, 10 in prospective designs, 7 in experimental studies, and 2 in mixed designs. Regulations were assessed with different instruments (53% with the *Behavioural Regulation in Exercise Questionnaire* (BREQ) and with Markland and Tobin’s revised version (BREQ-2) [37] and reported results in several ways: Relative autonomy was evaluated as a composite score (e.g., the *Relative Autonomy Index* (RAI), by which individual regulations are weighted and summed to give an index of the extent to which a person’s behavior is more or less autonomously regulated) in 23% of the cases (none of which were experimental designs); autonomous and controlled regulations were grouped and analyzed as two higher-level types of regulation in 21% and 14% of the cases, respectively. All major forms of regulation were assessed and discriminated in 71% of the cases.

**Figure 2:**
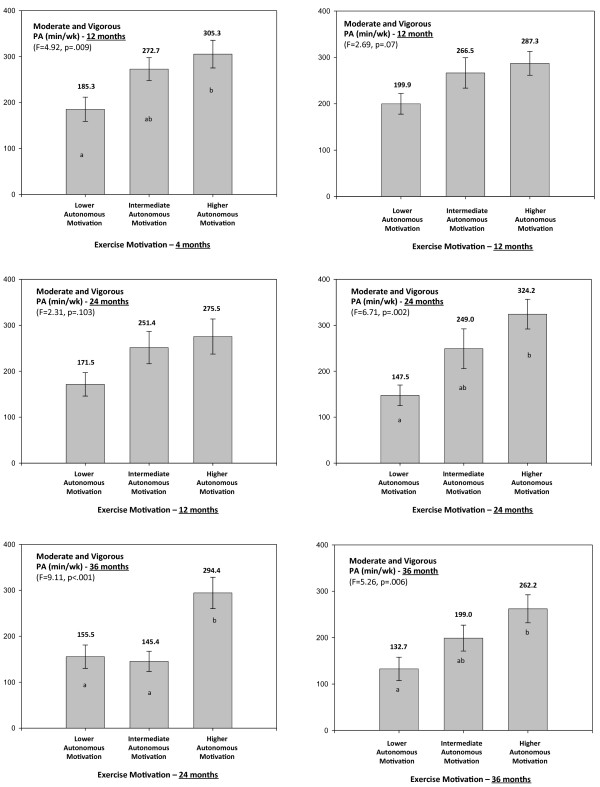
**Title. Self-reported minutes of moderate and vigorous exercise per week as a function of exercise autonomous motivation.** Analysis includes 141 participants of the PESO trial [67] and data reports to variables assessed at 12 months (intervention end), 24 months (1 year follow-up with no contact) and 36 months (2-year follow-up). The time-point values in exercise and motivational variables at each assessment period were used (not change). Values used for tertile-split groups of autonomous motivation were calculated including all subjects (intervention and control groups collapsed), adjusting for experimental group membership. Autonomous motivation includes the identified regulation and intrinsic motivation subscales of the *Exercise Self-Regulation Questionnaire*[84]. Self-reported exercise was assessed with the 7-day Physical Activity Recall interview [25] and quantifies moderate and vigorous structured physical activity (METs > 3) performed in the previous week (or typical of the previous month if previous week was atypical, see reference 27 for more details). Panels **B, D,** and **F** show cross-sectional associations (variables assessed at the same time point) and panels **A, C,** and **E** show “prospective” associations (motivation assessed one year earlier than exercise). F for one-way ANOVA with letters in bar indicating multiple comparisons with Bonferroni post-hoc tests (different letters indicate different means, p < .05).

Nearly all studies using measures of relative autonomy (8 of 9 *K*) reported positive associations with exercise behavior while studies investigating autonomous and controlled forms of regulation (*K =* 11 and *K =* 5, respectively) also found consistent, positive associations favoring autonomous regulations as a predictor of exercise outcomes (82/91%, depending on whether bivariate or multivariate analysis is used). On the other hand, 3 independent samples (60%) showed negative associations in multivariate models for non self-determined regulations, all others (40%) showing no association. In bivariate analyses, results for controlled regulations unanimously showed no association. Results were similar across different study designs, suggesting consistent positive effects of autonomous regulations on exercise behavior, and either negative or null effects associated with controlled regulations. In one study with longer-term follow-up measurements, prospective associations between regulations and exercise behavior were reported [33] (see also Figure 2). The authors found that both 12 and 24-month autonomous regulations, but not controlled regulations, mediated the effects of a SDT-based intervention on self-reported exercise at 24 months [32].

Specific results concerning the separate autonomous types of motivation showed positive associations between identified regulation and exercise behavior in 28 samples (74%) in multivariate analyses and 22 samples (85%) in bivariate analyses. The only exception was a study by Moreno et al. where the mean value for identified regulation was lower in a group reporting 60+ min of exercise than among those who exercised less than 60 min (presumably each day; no details are provided). Of note also are the mixed results found by Edmunds et al. (2007) displaying negative associations for identified regulations in a multilevel model, but positive cross-sectional associations at each of the 3 times points. The authors indicated that the multilevel results “should be ignored as they are a consequence of net suppression” [38]; pg.737]. In 3 studies that analyzed identified regulations [36,40,39], no significant association emerged. Regarding intrinsic motivation, positive associations with exercise behavior were reported in 23 or 22 independent samples (62% or 92%), in multivariate or bivariate analyses respectively. No study reported negative associations and results were consistent independent of study design. Few studies have tested the role of integrated regulation, but it appears to positively predict exercise behavior. Of 8 samples analyzed, 62-75% found positive associations with physical activity, with increased consistency found in bivariate analyses.

In an attempt to further clarify which single self-determined type of motivation is more closely related with behavior outcomes, a comparative analysis between identified and intrinsic motivation findings was undertaken. Twenty-five studies (*K =* 31) reported significant associations for both variables, of which 12 *K* were derived from multivariate analysis, 5 *K* from correlational analysis, and 4 *K* from both types of analysis. Seven studies (*K =* 7) found associations for identified regulation in multivariate analysis, but only bivariate associations for intrinsic motivation [44,45,43,42,41]. Three studies/samples showed the converse [48,47,33], reporting associations for intrinsic motivation in multivariate analysis and only correlational bivariate associations for identified regulation. It should be noted that no study tested whether the differences between the association coefficients (for identified regulation vs. intrinsic motivation) with exercise were significant. Wilson et al. (2002) investigated bivariate predictors of different physical activity intensities [49] and found that at mild intensities, associations were significant only for identified regulation; for moderately intense and strenuous exercise, both identified regulation and intrinsic motivation were significant predictors. Three additional studies/samples showed significant associations only for identified regulation [50,51,38]. In another study (*K =* 1) this regulation was the only variable predicting fewer exercise relapses [52]. On the other hand, two studies found significant associations only for intrinsic motivation [54,53].

For integrated regulation, only 6 studies (*K* = 8) were available. Comparing results for integrated versus identified regulations no differences were found in the patterns of association for all but one study [85] where there was a significant bivariate association with exercise for integrated but not identified regulation. Comparing results between integrated regulation and intrinsic motivation, two studies show integrated regulation, but not intrinsic motivation, as a significant predictor of exercise in multivariate models [41,38] whereas in a different study the opposite trend was observed using bivariate associations [28].

All studies measuring stages of change for exercise participation (*K =* 7) showed that autonomous regulations increased across stages, being the highest in the action/maintenance stages. However, only one study formally tested differences for regulations’ means across stages of change [52]. They found that for identified regulation there was a progressive increase from preparation to action to maintenance stage (ANOVA F = 25.1, p < 0.001) whereas for intrinsic motivation, maintenance had significantly higher means than both preparation and action stages (F = 27.5, p < 0.001). Five of these studies used the BREQ/BREQ-2 and 4 of these used discriminant function analysis. In these 4 studies, identified regulation loaded slightly stronger than intrinsic motivation on the primary discriminant functions distinguishing across stages of change. Authors tended to conclude that identified regulation played a more important role in exercise adherence when the full range of stages of change is considered. Finally, in a study examining change in behavioral regulations among exercise initiates, Rodgers et al. showed that both identified and intrinsic motivation increased overtime and that, compared to regular exercisers, initiates’ levels of identified and intrinsic motivations remained below regular exercisers’ levels even after 6 months of physical activity [31]. Authors also concluded that identified motivation appeared to increase faster than intrinsic motivation in these early stages of exercise adoption [31].

Results from multivariate analysis concerning the controlled types of motivation showed negative associations between external regulation and exercise behavior in 16 independent samples (43%). The remaining samples (57%) showed no associations. The trend for the absence of an association between external regulation and exercise was more apparent in bivariate analysis (77%). Regarding external regulation across stages of change, results show that external regulation generally decreases across stages, being higher in the preparation/action stages than in the maintenance stage. Furthermore, when comparing genders, results suggest that among males external regulation is negatively associated with exercise in the latter stages of change (i.e., maintenance) whereas among female there is no association at this stage.

Regarding introjected regulation, multivariate analysis showed positive associations with physical activity in 11 independent samples (30%), 1 study (*K = 2*) found negative associations (5%) and all others showed no association (65%). Bivariate results pointed in a similar direction, but showed more positive associations (52%). Despite the positive associations with exercise behaviors, the strength of association for introjected regulation appears to be lower compared to self-determined types of motivation, as reported in several studies [e.g., [55,49]. A closer look into the way introjected regulation predicts exercise participation over time shows mixed findings. Rodgers et al. (2010) studied initiate exercisers and found significant, but small, increases in introjection overtime, noting that these changes occurred mainly in the early stages of exercise participation [31]. Increases in introjected regulation were also observed across stages of change in 5 of 7 independent samples, although these were only significant in one case [e.g., [52]. In contrast, Silva and colleagues showed that although introjected regulation was cross-sectionally associated with exercise at 12- and 24-month time points, 12-month regulation did not prospectively predict (nor did it mediate) 24-month exercise outcomes [33,32].

A possible gender effect might be relevant to understand these mixed findings regarding introjected regulations. In effect, a closer examination of all the studies that explored gender differences with respect to the association between exercise regulations and behavior suggests that introjected regulation may be more positively associated with exercise among females, whereas among males the association is negative or zero [e.g., [45,41]. Within the studies examining differences across stages, results suggest that introjection is relevant for both genders in the action stage, but that in the maintenance stage it is more relevant for women than for men [56,55]. It should be noted that only two studies reported associations for men: one showed a positive association in the action stage and negative in the maintenance stage [55] and another study showed a tendency towards a positive association in the action/maintenance stage [57]. For studies with mixed samples and not reporting gender differences (the majority) the associations are mixed. Experimental studies confirm this pattern of mixed results, some showing increases in introjected regulation over the course of an exercise program [e.g., [39] and some showing no significant changes [e.g., [58]. One notes that null or unreliable results from introjection are theoretically expected within SDT, in which introjection is seen as an unstable basis for motivation without positive long-term utility.

Regarding amotivation, 5 independent samples (36%) showed negative associations with exercise outcomes in multivariate analysis; the remaining studies (*K =* 9) showed no associations. Correlational analysis showed negative associations in 9 samples (69%) and no association in 4 samples (31%).

*Need satisfaction.* A total of 17 samples/studies were used to analyze the associations between basic psychological needs and exercise behavior. Ten samples were evaluated in cross-sectional designs, 3 within prospective studies and 3 in experimental designs. One study used mixed methods (cross-sectional and prospective). Different instruments were used to assess basic needs, a fact that does not facilitate the comparison of results between studies. *The Psychological Need Satisfaction for Exercise Scale*[16] was adopted in 24% of the cases and was the most frequently used measure. Competence was assessed in 14 (82%) independent samples, autonomy in 11 (65%) samples, and relatedness in 9 (53%) independent samples. An examination of the specific multivariate results for each basic need showed that perceived competence was positively associated with physical activity in 56% of the independent samples, while the remaining samples showed no association (44%). The pattern of association was much clearer in correlational analysis with 12 samples (92%) reporting positive associations. Regarding autonomy need satisfaction, findings were mixed and generally ranged from no association (60% in multivariate analysis) to moderate positive or negative associations (20% for each). Nevertheless, positive correlations were reported in 5 studies/samples (50%) using bivariate analysis. Regarding relatedness, multivariate results consistently reported an absence of associations with exercise behavior (*K =* 4, 100%). Correlations showed a similar pattern, even though a general trend towards a positive association with exercise behavior was identified (38%). No negative associations with exercise outcomes were observed for the perceived fulfillment of any of the 3 needs. A composite score was created to assess overall exercise psychological need satisfaction in 2 (of 17) samples; positive associations with exercise behavior were reported in both cases.

*Exercise motives.* A total of 12 studies (*K =* 15) investigated the associations between motives (or goal contents) and exercise behavior. Of these studies, 8 were cross-sectional, 3 prospective, and 1 used a mixed design (cross-sectional and experimental). Regarding the instruments used to measure exercise motives, there is some inconsistency: the *Motives for Physical Activity Measure* (MPAM) or MPAM revised/adapted versions [59,27] of it were used in 6 independent samples (40%), 3 samples (20%) measured exercise motives using the *Exercise Motivations Inventory - 2* (EMI-2) [60], and in other 3 samples (20%) the *Intrinsic Motivation Inventory* (IMI) [61] was employed to evaluate intrinsic motives and the *Extrinsic Motivation Inventory* (Lee’s EMI) [62] to measure extrinsic motives. Sebire and colleagues (2009) [19] used the recently developed *Goal Content for Exercise Questionnaire*[63] while Segar and colleagues used an inductive, qualitative method to assess exercise motives in one study [64], and performed a cluster analysis to identify homogeneous groups of goals, intrinsic and extrinsic, in another study [65].

Multivariate results showed that intrinsic motives (e.g., challenge, affiliation, enjoyment) were positively associated with exercise behavior in all samples (*K =* 8, 100%). A similar trend was observed in correlations (75%). Regarding body-related motives, multivariate findings were mixed regardless of the statistical analysis performed: in multivariate analysis, 25% of the samples showed positive associations and 25% reported negative associations; in correlational analysis, a general trend towards a positive association was identified (63%). The pattern of association was less clear for health/fitness motives with 33% showing positive associations, 33% showing negative associations, and other 33% not finding any association. There was only one study/sample performing correlational analysis to explore the links between health motives and exercise [46]; positive associations were reported. As expected from theory, controlled motives (social recognition, appearance/weight) did not predict, or negatively predicted, exercise participation [46].

*Perceived need support.* Environments perceived as more need-supportive were positively associated with increased levels of self-reported physical activity in 3 (of 6) independent samples tested with multivariate analysis (50%). This increased to 73% (*K =* 8) in correlational analysis. The remaining studies/samples showed no association. In the majority (67%) of independent samples perceived need support was assessed using the Health Care Climate Questionnaire [66].

*SDT-based Interventions.* To date, only a few interventions have been designed to promote exercise-related behaviors by specifically increasing personal autonomy in the form of exercise autonomous self-regulation in adults [e.g., [17,40,68,39,67,69]. Some of these trials are still ongoing and all have been conducted in Western cultures. Of 7 interventions (with available data), 6 (86%) found significant differences favoring the SDT-based intervention group for perceived autonomy support, need satisfaction, and autonomous and introjected regulations for exercise, as well as greater self-reported exercise. In addition, one of these interventions found gender differences, reporting significant increases in perceived autonomy support and self-reported exercise only for women [40]. In contrast, there was one study in a clinical setting that did not find significant differences in perceived autonomy support and exercise behavior between autonomy support group and controls [68]. The authors argued that their additional individual SDT-based 4-week intervention, added to standard cardiac rehabilitation, might have been too limited (i.e., an insufficient number of sessions) to achieve significant between-group differences.

Edmunds and colleagues tested a SDT-based intervention in an exercise setting, examining the effect of an autonomy-supportive teaching style on female exercisers’ psychological needs, motivational regulations, and exercise behaviors during a 10-wk exercise program [39]. They found that the intervention increased autonomous self-regulation, need satisfaction, and attendance [39]. Although not a randomized controlled trial, results were similar to those obtained in several RCTs. For instance, Fortier et al. [17] tested an autonomy-promoting counseling protocol for promoting physical activity in sedentary primary care patients in a 13-week RCT. Results showed that the intervention was successful in changing autonomous self-regulation to reach activity goals (vs. a brief counseling protocol) and that higher autonomous regulation for exercise mid-intervention predicted higher levels of physical activity at the end of the intervention in the intervention group. The longest RCT to date to evaluate autonomy support, need satisfaction, motivation, and exercise behaviors was implemented in 239 overweight women, through 30 weekly group sessions for about 1 year, with a 2-year follow-up [67]. A few features of this study clearly distinguish it from the remaining intervention studies reviewed (see Table 2, table VI): larger sample, considerably longer intervention and follow-up assessments up to 3 years, and the use of mediation analysis to predict long-term changes in physical activity. Results showed that the intervention was perceived as need-supportive, it increased perceptions of competence and autonomy for exercise, increased autonomous regulations (and to a lesser degree introjected regulation, but not external regulation), and increased exercise behavior [18]. Exercise level was clearly associated with level of autonomous motivation for all subjects, both concurrently and prospectively, as depicted in Figure 2. Only autonomous regulations were found to mediate the intervention effect on exercise in the long-term [33,32].

## Discussion

The aim of this review was to examine the empirical literature on the relations between SDT-based constructs and exercise and physical activity. The review demonstrates the recent growth in the application of this theory to the study of exercise and physical activity motivation, with 53 of the 66 papers identified being published in the last five years. The theory has been applied to a wide range of physical activity contexts including recreational exercise, weight loss programs and clinical populations, and across a range of ages. The majority of studies employed cross-sectional designs but comparable results are found across cross-sectional, prospective, and experimental designs.

### Behavioral regulation and exercise

The vast majority of studies included an examination of the relations between behavioral regulation and exercise behavior. Of these, most included some or all of the individual regulations specified within SDT whereas others have collapsed autonomous and controlled forms of regulation into summary scales or adopted the RAI. The results show consistent support for a positive relation between more autonomous forms of motivation and exercise behavior, whether single regulation, summary measures, or the RAI are used. Intervention studies are also clearly supportive as are studies examining the endorsement of different forms of behavioral regulation across the stages of change, consistently showing that more self-determined regulations distinguish between individuals in the later stages from those in the early stages.

When considering the more autonomous forms of behavioral regulation separately, positive associations for identified regulation are found slightly more consistently in comparison to intrinsic motivation in multivariate analyses, whereas intrinsic motivation is somewhat more consistently predictive of exercise behavior in bivariate analyses. A similar trend was found for integrated regulation versus intrinsic motivation, but based on much fewer studies. This could be interpreted as suggesting that, *independent of* other regulatory motives, identified regulation (or integrated regulation) is the single best correlate of exercise. This notwithstanding, the SDT continuum of motivation [10] suggests that regulations that are more closely located in the continuum of autonomy specified by SDT (such as identified and integrated regulation, and intrinsic motivation) are expected to share some degree of variance, highlighting the theoretical expectation that regulatory factors are often simultaneously operative. This renders the question of which sub-type of autonomous motivation is more important in explaining and promoting exercise behaviors difficult to solve. Nonetheless, a number of authors have discussed this issue, attempting to explain results “favoring” either identified or intrinsic motivation. For example, Mullan et al. [57] argued that intrinsic motivation alone is unlikely to sustain long-term regular engagement in exercise, given all the organization and commitment it entails. Edmunds et al. [44] suggested that because sustaining a physically active lifestyle presumably requires a high degree of effort, often for mundane or repetitive activities, regulation by identification with the outcomes may be more important than exercising for fun and enjoyment, or to challenge oneself. Finally, Koestner and Losier (2002) proposed that in behavioral domains that require engagement in a range of different activities that vary in their intrinsic appeal, internalization of the value of the outcomes of the activities is likely to lead to greater persistence than being intrinsically motivated [70]. Clearly exercise is one such behavioral domain.

Because health promotion campaigns typically market exercise more in terms of health-related outcomes than in terms of its intrinsic value, the primary source of self-determined motivation among active individuals might derive from a valuing of these outcomes, even if they also find exercise intrinsically enjoyable [55]. Conversely, in contexts where enjoyment in and genuine interest for exercise is emphasized over the outcomes, one might expect intrinsic motivation to be more salient to individuals. In support of this, in Silva et al.’s intervention that explicitly emphasized enjoyment, mastery and challenge rather than the outcomes of exercise, intrinsic motivation was a more consistent predictor than identified regulation of moderate and vigorous exercise [33]. Clearer definitions of the nature of the exercise behaviors under investigation (type, intensity, volume, duration, time in the same activity), which may vary within and among studies, and their potential appeal to the individual may shed additional light onto this issue. Some types of physical activity may be inherently intrinsically motivating for many individuals, especially when they involve self-chosen optimal challenges that can help people enjoy the sense of autonomy and mastery, factors that underpin intrinsic motivation.

As Daley and Duda [55] point out, most of the research showing a stronger effect for identified regulation has been cross-sectional and a few studies, including experimental studies lasting for several years, have shown intrinsic motivation to be critical for longer-term engagement [44,32]. Furthermore, a major limitation in interpreting findings concerning a benefit for either identified regulation or intrinsic motivation is that where associations for both have been found, authors have not conducted statistical tests to determine the unique effects of each type of regulation, nor whether the larger effect is in fact statistically significant. Given also the lack of longitudinal or experimental studies to determine whether differential benefits for the two types of regulation might emerge over time, it would be advisable for the time being to recommend fostering *both* identification and intrinsic motivation in order to promote optimal behavioral outcomes. Both of these autonomous forms of motivation share common antecedents in terms of support for autonomy and competence. Identification could be specifically promoted by emphasizing the personal instrumental value of exercising with regard to health, optimal functioning, and quality of life. At the same time, intrinsic motivation could be promoted by emphasizing fun, skill improvement, personal accomplishment, and excitement while exercising. Furthermore, the focus should be not only on the amount of exercise performed, or long-term adherence *per se*, but also on the enhanced well-being and vitality associated with exercise. Indeed, intrinsic motivation has been shown to be not only related to persistence at a task but also with psychological health and improved well-being [15].

The results for more controlled forms of regulation are mixed. No studies have found a positive association for controlled motivation at the summary level of analysis, nor for external regulation at the individual regulation level. However, while a substantial number of studies found a negative association, the majority found no association. There is a trend for external regulation to be negatively associated with exercise in the later stages of change among males, but no association among females, suggesting that more active males might respond more negatively to social pressures to exercise.

Concerning introjected regulation specifically, results are split between positive and null relations with exercise, with a clear predominance of the latter in multivariate analyses. This internally controlling form of regulation is generally theorized to be associated with more maladaptive outcomes such as negative affect, feelings of guilt, and lowered self-esteem [12]. People who feel internally pressured to exercise are likely to experience some degree of guilt or shame if they do not exercise, and the potential to enjoy it and experience the positive well-being consequences of this behavior will be decreased. Furthermore, research examining the motivating forces behind exercise dependence, which is considered to be maladaptive, has found introjected regulation to be the strongest predictor of this type of dependence [51]. Nonetheless, the periodic finding of a positive relation between introjection and adaptive behavioral outcomes in both exercise and other behavioral domains has been attributed to the partial internalization of external pressures from, for example, health promotion messages [52] or parental expectations [71].

When energized primarily by introjected motives, exercise participation may occur at some cost to psychological health, a factor most exercise adherence studies have not quantified. By contrast, recent evidence in overweight women showed that a summary measure of controlled exercise regulation (including introjected and external regulation items) was unrelated to psychological well-being, although controlled motivation to participate in obesity treatment predicted lower quality of life and self-esteem, and higher state anxiety [72]. A more refined analysis of introjected forms of motivation, breaking it into an approach-orientated motivation (to seek positive feelings such as self-aggrandizement and pride) and an avoidance-oriented motivation (to avoid negative feelings such as shame, guilt, and anxiety) could help clarify the role of introjected regulation on psychological and possibly also on behavioral outcomes [20]. Introjected avoidance regulation has been shown to yield more negative psychological correlates, including less engagement in school or poorer sports performance than introjected approach regulation [73]. The former was also more strongly associated with identified regulation than the latter. To our knowledge, studies have not yet addressed the differential association of these subtypes of introjected regulation with exercise behavior adoption or persistence.

The studies reviewed here also show a trend for an increase in introjection over time in the longitudinal or experimental studies, or across stages of change. However, observed (or assumed) increases in introjection with time do not necessarily mean that this variable explains or mediates increases in exercise. For instance, introjection has been found to be significantly associated with exercise when both were measured at the same time point, but not prospectively [32], suggesting that regulation by introjection may not lead to sustained exercise behavior. Furthermore, and despite observed increases in introjected regulation as a result of an SDT-based intervention [18], only autonomous motivation was predictive of long-term moderate and vigorous exercise in mediation analysis [32]. Unfortunately, there is only one study [32] reporting such long-term prospective associations between experimentally-induced changes in motivation and exercise behavior.

Our analysis of the relation between introjection and exercise for those studies reporting associations separately for males and females provides some evidence for a gender effect. Where such effects occur, introjection appears to be more positively associated with exercise among women, whereas among men there is a negative association or no association, especially in the maintenance stage of change. Some studies also report no differences. Given the pervasive societal and media pressures on women to have a slim and toned physique [74], this is perhaps not surprising. In the majority of studies, gender differences are not reported, making it difficult to draw firm conclusions but the trends we observe here for both introjection and external regulation suggest that future research would do well to consider possible gender differences rather than assuming no such differences and collapsing data across gender.

Finally, with regard to behavioral regulations and exercise, unsurprisingly no studies found a positive association between amotivation and exercise. The remaining studies showed either a predominance of null findings (nearly 70% in multivariate analyses) or negative associations (64% in bivariate analyses). Closer examination of these studies shows a trend for a sample effect. In all five studies showing no association the samples comprised either non-exercisers or a mixture of non-exercisers and exercisers, while the majority of studies showing negative associations comprised regular exercisers. Furthermore, it is noteworthy that fewer studies have assessed amotivation in comparison to those assessing the other regulations. This is understandable given that amotivation refers to the absence of both intrinsic and extrinsic motivation and represents a complete lack of self-determination and volition with respect to the target behavior [12]. Therefore one would expect to rarely see highly amotivated individuals in exercise settings. Additionally, different authors have put forth the hypothesis that individuals could also be autonomously motivated to *not* participate in exercise upon consideration, perhaps even when they can perceive some value in the behavior [7,20]. In some respect, they would be “autonomously amotivated” towards exercising. To the extent this would occur, it might also confound the association between amotivation and exercise, since these individuals might not score high on typical amotivation items such as “I don’t see the point in exercising” and “I think that exercising is a waste of time”, despite behind sedentary. It should also be noted that, empirically, it is difficult to distinguish amotivation from a lack of controlled or autonomous regulation [46]. Hence, including amotivation along with controlled and autonomous regulation in the same model might introduce a confound and could help explain the absence of associations in multivariate analyses.

### Need satisfaction and exercise

Rather less attention has been paid to examining the associations between satisfaction of psychological needs and exercise than for behavioral regulations. The use of different instruments to assess basic need satisfaction (both domain-general and domain-specific measures), differences in the number of needs assessed, and their combined or separate analyses do not facilitate easy comparison of results across studies. Generally, competence satisfaction has been the most frequently assessed need and the literature shows consistent support for a positive association with exercise. In this review, twice as many studies reported bivariate associations between need satisfaction and exercise, compared to multivariate analyses. In bivariate analyses, no studies report a negative association between autonomy and exercise and the remaining results are split equally between positive and null associations whereas multivariate results are more mixed. Results for relatedness satisfaction are also mixed in bivariate analyses, although again no studies found a negative association with exercise. The exercise context might explain a lack of association for relatedness satisfaction. In some contexts, engaging in solitary exercise being the most obvious, the need for relatedness might simply not be an issue. Inconsistency in the measures used to assess the needs, and therefore their operational definitions, and a lack of applicability of particular scales to different exercise contexts might be concealing positive associations for autonomy.

In interpreting the results for need satisfaction and exercise, it is important to note that only direct effects of need satisfaction on exercise (whether from bivariate or multivariate association or direct paths in structural models) were considered in the present review, a fact that does not consider their indirect effects. In fact, theorizing within SDT stresses that the internalization of behavioral regulations is fostered by the satisfaction of basic psychological needs, and thus autonomous regulations would mediate associations between need satisfaction and behavioral outcomes. In current interpretations of mediation analysis, a significant association between an independent and a dependent variable is not a necessary condition for the possible occurrence of significant indirect (i.e., mediated) effects between them [75]. This highlights the importance of conducting more sophisticated analyses, such as path analysis or structural equation modeling, to clarify the mediating role of need satisfaction in the development of self-determined motivation. Indeed, going beyond the simple direct associations between behavioral regulations or need satisfaction and exercise (which are the main focus of this review), it is important to note that several studies have tested one or more parts of SDT’s proposed motivational sequence(s) for physical activity behaviors (see Figure 1). Relations from perceived autonomy support to exercise behavior, via psychological needs and regulatory styles have been tested (in part or all) in several studies and in general these confirm the proposed sequences [17,44,43,77,76,38,33]. In one case this was tested with a longitudinal randomized controlled trial using structural equation modeling [33,32], which empirically supported the motivational sequence proposed by SDT (i.e., need-supportive health care climate - > need satisfaction - > autonomous exercise regulation - > exercise behaviors).

### Participation motives and exercise

Following some early work in the 1990s, there has been a resurgence of research in recent years on the role of exercise participation motives or goal contents. The rationale for this is that some motives (e.g., affiliation, skill development) are more intrinsically-oriented and likely to be experienced as autonomous whereas others (e.g., body-related motives such as weight or appearance management) are more extrinsic and likely to be experienced as internally controlling. Studies show a consistent positive association between more intrinsic motives and exercise. Findings for fitness/health and body-related motives are mixed. For fitness/health, although no studies found a negative association, an absence of association is more frequently found than positive associations. This might reflect different ways in which fitness/health motives have been operationalized. Health/fitness motives can reflect health pressures or threats (e.g., medical advice) or be associated with drives for thinness or an attractive image. Yet health and fitness motives can also reflect more positive concerns such as general health promotion, increasing physical strength for performing daily activities, reducing pain (e.g. lower back pain or discomfort in joints), or feeling more energy and vitality. Thus, conceptually, being concerned about health or fitness *per se* cannot be easily defined as either intrinsic or extrinsic, as it depends on what the motive means to the individual [78].

Similarly, results for body-related motives results are also mixed, despite a preponderance of both positive and null findings, relative to negative associations. For a more in-depth understanding of the relation between participation motives and exercise, the characteristics of exercise participation (e.g. type, intensity, total volume) and type of sample need to be taken into account. For example, Frederick and Ryan (1993) compared individuals whose primary physical activity was a sport with individuals whose primary physical activity was a non-sport fitness activity [59]. The sport participants had higher interest/enjoyment and competence motives whereas the fitness participants had higher body-related motives. Furthermore, the apparent positive (at least in the short term) role of these motives on exercise may then be mediated by the development of introjected regulation. Ingledew et al. [79,46] found that body related motives were associated with introjections and a recent study [41] found that introjected regulation predicted exercise intensity among females.

 It is important to note, as Markland and Ingledew pointed out [46], that holding controlled motivations is not necessarily problematic, motivationally speaking, as long as self-determined regulations are also held. It has been suggested [20], for example, that a person may strive for a physically appealing body (an “extrinsic” motive) because her partner praises her good looks (controlled motivation) and at the same time she may personally value a fit appearance (autonomous motivation). Thus, although intrinsic goals tend to be pursued for autonomous reasons and extrinsic goals tend to be pursued for controlled reasons [81], the content of, and reasons for pursuing aspirations can be empirically crossed. Therefore, exercise promotion programs should take care not to explicitly or implicitly denigrate appearance/weight motive or any other motive for exercising, which may lead individuals to perceive that their autonomy is threatened, with consequent defiance and dropout [46]. Instead, acknowledging the validity of individuals’ motives in a need-supportive context may ultimately promote movement away from controlled regulations toward more autonomous commitments to be active.

### Experimental studies

It is encouraging to see that in more recent years researchers have turned their attention to experimental studies evaluating interventions based on SDT principles. However, all but one were shorter than 3 months in duration and involved a small amount of contact time with the participants, in some cases amounting to approximately 2–3 in-person sessions. The remaining contacts were performed via telephone [e.g., [17,68,69], and one of these interventions relied solely on email booster messages to promote self-determined motivation and behavior change [40]. By contrast, one intervention provided substantially more contact time, (thirty 2-hour group sessions for about 1 year [18,67]). Not surprisingly, intensity, depth, and strategies used to promote personal autonomy and the development of intrinsic motivation for exercise also varied among these interventions. Some interventions were limited to strategies such as encouraging participants to make their own choices, providing information, setting realistic goals, and/or encouraging participants to seek and find forms of social support [e.g., [17,40]. Others included a more comprehensive set of strategies, more fully embracing SDT propositions [18,39,67] including providing a clear rationale for behavior change, acknowledging ambivalence and internal conflict, providing a menu of options, minimizing controlling influences (e.g., use of pressure, demands, and extrinsic rewards), and promoting competence through optimal challenge and giving informative feedback [18,33,32]. In sum, existing interventions are limited in number and highly varied. Longer and more comprehensive longitudinal interventions are needed, especially those which work toward the development of autonomous motivation, allow more time for changes in motivational and behavioral processes to take place, and assess whether those changes (and associations) persist in the long-term.

## Conclusions

Overall, this review provides good evidence for the value of SDT in understanding and promoting exercise behavior. The clearest finding of this review concerns the beneficial role of developing autonomous self-regulation, be it predominantly via autonomous forms of extrinsic regulation (i.e., identified and integrated regulation) or enhanced intrinsic motivation. The present literature is consistent in showing that all forms of autonomous regulation predict exercise participation across a range of samples and settings. There is also increasing evidence that a motivational profile marked by high autonomous motivation is important to sustain exercise behaviors over time, although the pool of studies supporting this inference is limited. Longer-term studies and follow-ups will be especially important in evaluating the relative efficacy of identified versus intrinsic regulations in exercise maintenance. For the moment, evidence is consistent with the hypothesis that reporting well-internalized extrinsic regulations, such as personally valuing certain *outcomes* of exercise, is a particularly important factor for initial adoption (when cognitive factors such as rationally weighing pros and cons may be decisive but experiential knowledge of exercise may be limited). Conversely, there is some indication that a predominance of intrinsic motivation (i.e., valuing the actual *experience* of exercise) is especially important for longer-term exercise participation. It is also important to highlight the strong co-variance between identified/integrated regulations and intrinsic motivation, especially since these different forms of autonomous motivation share some common antecedents that would be applied in intervention settings.

We suspect future studies may come to identify significant moderating factors for the role of specific regulations on exercise adherence, such as age, gender, previous health conditions, or social norms and social desirability. For instance, current public campaigns against obesity may have enhanced the perceived *utility* of exercise for weight control and health (as a preventive or treatment “medicine”), inadvertently minimizing experiential rewards of exercise such as social interaction, expression of personal skills and abilities, self-development, or pure enjoyment. The experiential qualities of exercise were highlighted as a critical factor for adherence in a recent review of mediators of physical activity behavior change [82]. On this note, it is perhaps no coincidence that in the current public health dialogue about “exercise as medicine”, physical activities not typically associated with the term “exercise” such as playing sports, dancing, or outdoor exploration activities are rarely mentioned. From a public health/exercise promotion perspective, this could be a limiting factor if such activities, rich in their intrinsic appeal although less likely to be monitored and supervised, are not considered viable options in professionals’ exercise prescriptions or as targets of public policy promotions. Again, future research with long-term outcomes and also exploring predictors of different forms of exercise should help elucidate these issues.

Two additional conclusions can be derived from the present review. One is that having more intrinsic participation motives or goals associated with exercise, such as affiliation and social engagement, challenge, and skill development, is clearly associated with greater exercise participation. Since these motives are associated with intrinsic motivation [22,34], it may be especially important that health professionals are trained in distinguishing the “signs” of intrinsic (vs. extrinsic) motives in their patients and promoting them at every opportunity, aiming at long-term exercise maintenance. The other is that reporting increased perceived competence for exercise is also positively predictive of more adaptive exercise behavioral outcomes. Together, the previous findings have important implications for practice. It serves as evidence-based support for health professionals to strive not only to provide sufficient structure and optimal challenge to promote feelings of mastery and competence in their clients and patients, but also to encourage professionals to actively explore with the people they counsel reasons to be physically active that go beyond the most common motives such as improved body shape and attractiveness. Finally, as we discussed previously, the consequences of health and fitness-related motives, including weight loss, are perhaps more complex and likely moderated by other motivational aspects.

Limitations in the collective body of work are worthy of consideration as they bear on avenues for future research. A major limitation concerns the heterogeneity of the samples in the majority of studies. Heterogeneity within samples with regard to such factors as age, gender, weight or body composition, and fitness status may be contributing to variability across studies. While general motivational patterns are likely to remain constant (e.g., autonomous motivation being more likely to promote long-term exercise adherence), there may be much to learn by examining motivational profiles that are specific to different demographic groups or to individuals at different stages of change for exercise. For instance, a recent study [63] highlights the existence of different patterns of motivation between long-term exercisers versus beginners. Similarly, more enduring individual differences could be explored. Only one study has examined the relations between exercise causality orientations and exercise, and none have explored general causality orientations, despite the fact that such individual difference measures have been shown to predict adaptive outcomes in other health-related contexts [e.g., [108]. Finally, SDT has a history of strong experimental work on motivational factors but experimental work in the exercise domain itself could be expanded to better examine the causal mechanisms and process aspects of motivation for physical activity. Cross-sectional research is now abundant, and generally supportive, but it needs to be complemented with more applied intervention and translational studies that adequately model, implement, and evaluate key hypotheses about why and how individuals adopt and sustain more physically active lifestyles.

The methodology used in this review may also limit its conclusions. First, unpublished studies, evidence from grey literature, and data from non-English publications were not included. Although this is a frequent occurrence in scientific systematic review papers, it may provide an incomplete account of all studies in this area. Second, the way in which results from each study were classified and quantified (see Table 3) is somewhat arbitrary and subject to criticism and various interpretations. Third, as stated before, the decision to only evaluate direct paths is also inherently limiting considering that the distal effects of some variables on behavior is thought to be mediated by other intermediate variables. Unfortunately, few studies are available to assess these more complete causal paths. Finally, our definition of “behavioral variable” to describe the outcome of choice, lumping together self-report and direct measures of behavior, and also attendance and stages of change is clearly not without reproach. Although we felt this was the best decision considering the relative paucity of studies for various measures, future studies might want to be more specific and/or selective in their outcomes of choice.

In sum, it is clear that the exercise domain has provided fertile ground for testing SDT’s precepts. While testing and developing theory is a worthwhile activity in its own right, the real significance of SDT will be realized if it can be employed to actually make a positive difference in peoples’ lives. In this regard, the growing evidence for the utility of SDT-based interventions for promoting the adoption and maintenance of exercise is a significant advance. Future studies would do well to include biological markers of successful exercise-related outcomes such as increased fitness and reductions in disease risk factors. Similarly, studies that include markers of psychological well-being and mental health, such as self-esteem, vitality, and symptoms of anxiety and depression symptomatology would also be useful, given that according to SDT only autonomously regulated behaviors can translate into enhanced psychological wellness. Extending SDT´s applicability beyond behavioral engagement in exercise to actual improvements in health and well-being would thus be another important step for SDT research to influence health care policy and delivery.

## Endnotes

^a^Exercise outcomes covered in this review include what is normally termed “exercise” (purposeful and formalized leisure-time physical activity, often with the goal of improving fitness or health) but also, in a few cases, less structured forms of exercise (e.g., walking minutes), energy expenditure measures, and accelerometry data (which cannot distinguish between different forms of activity). Although the term “physical activity” would aptly cover the entire range of outcomes in this review, “exercise” is a more specific term to what the large majority of studies measured, with the use of instruments such as the Godin Leisure Time Exercise Questionnaires (LTEQ, used in 55 independent samples [77.5%]). For this reason, we will use the two terms indiscriminately in this review.

## Competing interests

The authors declare that they have no competing interests.

## Authors’ contributions

PJT conceived this manuscript and led the writing team. EVC conducted the study search, summarized the quantitative review, and drafted the Results section. DM made substantial contributions to the Discussion section. DM, RMR, EVC, and MNS revised the entire manuscript and made important contributions in various sections. All authors read and approved the final version of the manuscript.
